# A comparative analysis of *Danionella cerebrum* and zebrafish (*Danio rerio*) larval locomotor activity in a light-dark test

**DOI:** 10.3389/fnbeh.2022.885775

**Published:** 2022-08-04

**Authors:** Nina Lindemann, Leon Kalix, Jasmin Possiel, Richard Stasch, Tamia Kusian, Reinhard Wolfgang Köster, Jakob William von Trotha

**Affiliations:** Division of Cellular and Molecular Neurobiology, Zoological Institute, Technische Universität Braunschweig, Braunschweig, Germany

**Keywords:** *Danionella cerebrum*, *Danio rerio* (zebrafish), crystal, locomotor activity, light-dark test

## Abstract

The genus *Danionella* comprises some of the smallest known vertebrate species and is evolutionary closely related to the zebrafish, *Danio rerio*. With its optical translucency, rich behavioral repertoire, and a brain volume of just 0.6 mm^3^, *Danionella cerebrum* (*Dc*) holds great promise for whole-brain *in vivo* imaging analyses with single cell resolution of higher cognitive functions in an adult vertebrate. Little is currently known, however, about the basic locomotor activity of adult and larval *Danionella cerebrum* and how it compares to the well-established zebrafish model system. Here, we provide a comparative developmental analysis of the larval locomotor activity of *Dc* and *AB* wildtype as well as *crystal* zebrafish in a light-dark test. We find similarities but also differences in both species, most notably a striking startle response of *Dc* following a sudden dark to light switch, whereas zebrafish respond most strongly to a sudden light to dark switch. We hypothesize that the different startle responses in both species may stem from their different natural habitats and could represent an opportunity to investigate how neural circuits evolve to evoke different behaviors in response to environmental stimuli.

## Introduction

With just 10–15 mm in body length, the genus *Danionella* comprises some of the smallest known extant vertebrate species (Roberts, 1986; Britz et al., 2021). The natural habitats of these miniature cyprinids are the slow-flowing and rather shallow but turbid streams of southern Myanmar and north-eastern India (Roberts, 1986; Britz et al., 2021) that are also home to other members of the subfamily of Danioninae, possibly including the zebrafish, *Danio rerio* too (Parichy, 2015). In fact, phylogenetic analyses have shown that *Danionella* are closely related to the zebrafish as they form a sister group of the genus *Danio* and diverged from a common ancestor about 36 million years ago (Britz et al., 2009; Tang et al., 2010).

Consistent with its miniature body plan, the adult brain of *Danionella cerebrum* (*Dc*) [previously erroneously described as *Danionella translucida* (Britz et al., 2021)] has a volume of just 0.6 mm^3^ and consists of approximately 6.5 × 10^5^ neurons (Schulze et al., 2018) whereas the adult zebrafish brain has a volume of 2.8 mm^3^ (Kenney et al., 2021) and consists of approximately 1.0 × 10^7^ cells (Hinsch and Zupanc, 2007). Recently, it has been shown that *Dc* is amenable to transgenesis, and remains optically translucent during adulthood, particularly in the *tyr* background (Schulze et al., 2018), thereby enabling the application of whole-brain *in vivo* imaging techniques (Penalva et al., 2018; Schulze et al., 2018). Larval but not adult zebrafish, particularly in the *crystal* background (Antinucci and Hindges, 2016), are also optically translucent, and have a brain volume of less than 0.5 mm^3^ that is made up of 1.0 × 10^5^ neurons (Randlett et al., 2015). The zebrafish larval brain is therefore only slightly smaller than the adult *Danionella* brain, which is why thus far larval zebrafish have been in the vanguard of whole-brain *in vivo* imaging analyses (Ahrens et al., 2013; Ahrens and Engert, 2015; Vanwalleghem et al., 2018). However, with their yet immature brains previous studies suggested that larval zebrafish were largely lacking behind their juvenile and adult counterparts in performing associative learning tasks, or emotional and social behaviors thereby pointing toward the possibility that the underlying neural circuits enabling higher cognitive functions and behaviors may not be fully developed and functional at this early developmental stage (Valente et al., 2012; Dreosti et al., 2015; Huang et al., 2020; Stednitz and Washbourne, 2020). More recently though this notion has been challenged as it has been shown, for example, that the performance of 7 days post fertilization (dpf) larval zebrafish equals those of juveniles (21 dpf) and adults (90 dpf) in a spatial discrimination task (Santacà et al., 2020a,b), that 7–10 dpf larvae appear to be capable to learn active avoidance in an operant conditioning task (Yang et al., 2019), or that 10–12 dpf zebrafish can be trained to associate different colors and geometric shapes with a food reward in an appetitive learning task (Santacà et al., 2022). *Danionella*, therefore, holds great promise for future investigations of whole-brain *in vivo* imaging analyses of higher cognitive functions and behaviors, in particular during adulthood but also throughout its entire life cycle.

As an emerging neurophysiological model system, little is currently known, however, about the basic locomotor activity of *Danionella* and how it compares to the well-established zebrafish both at the larval and adult stage. Furthermore, little is currently also known about the larval locomotor activity of the optically translucent pigmentation mutant *crystal* that is particularly suited for whole-brain *in vivo* imaging analyses, since it offers an unique access to the forebrain in light sheet microscopy (Antinucci and Hindges, 2016), whereas the locomotor activity of other zebrafish strains, including the pigmentation mutant *casper* (White et al., 2008), in which forebrain structures are largely inaccessible by conventional light sheet microscopy (Antinucci and Hindges, 2016), has been characterized previously (de Esch et al., 2012; Lange et al., 2013; van den Bos et al., 2017; Audira et al., 2020). Developed for zebrafish larvae, the light-dark test is a high-throughput behavioral paradigm that can be used to analyze locomotor activity during alternating light/dark periods in multi-well plates (Prober et al., 2006; Emran et al., 2008; MacPhail et al., 2008; Irons et al., 2010; Padilla et al., 2011; de Esch et al., 2012; Brun et al., 2019; Fitzgerald et al., 2019; García-González et al., 2021). Locomotor activity of wildtype zebrafish in the light-dark test follows a standardized pattern during both the light and dark periods that can be classified into three phases (Emran et al., 2008; MacPhail et al., 2008; García-González et al., 2021). Immediately after the switch from light to dark, (i) zebrafish larvae respond with a startle response, they then (ii) increase their velocity relative to preceding light period (and higher than baseline level) before (iii) decreasing their velocity again (still higher than baseline level). After the switch from dark to light, (i) zebrafish larvae also respond with a startle response, then (ii) decrease their velocity relative to the preceding dark period [and lower than baseline level (freezing)] before (iii) increasing their velocity again (equal to baseline level). Here, we made use of the light-dark test to analyze and characterize the larval swimming behavior and locomotor activity of 4–6 dpf *Danionella cerebrum* and compare it with *AB* wildtype and *crystal* zebrafish. We found similarities but also differences in both species.

## Results

**FIGURE 1:**
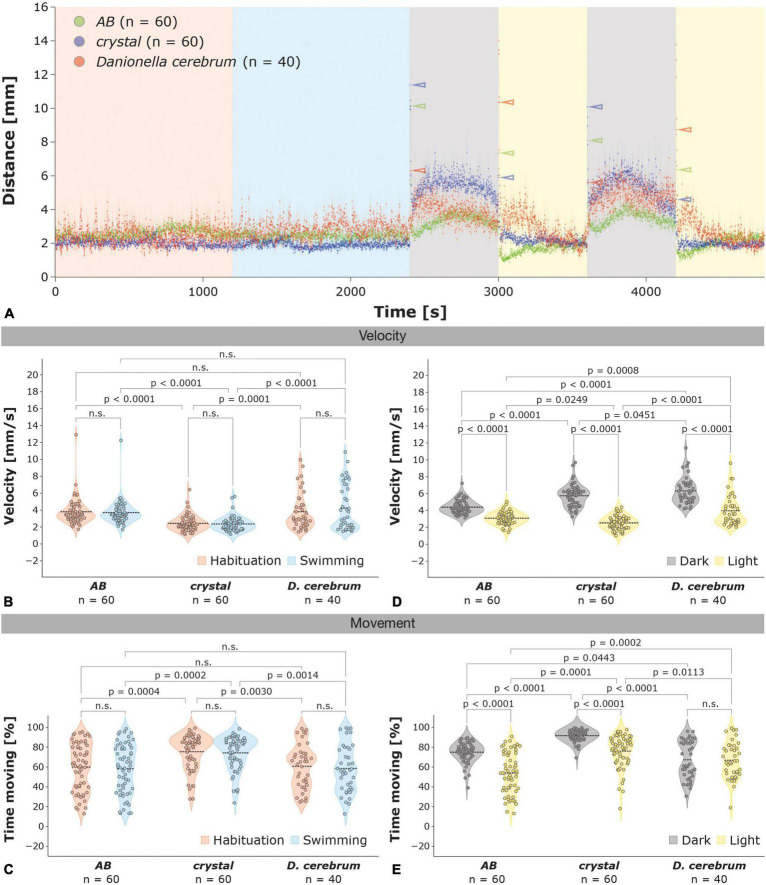
Locomotor activity of 6 dpf zebrafish and *Dc* larvae in the light-dark test. **(A)** The light-dark test consists of a habituation (20 min; orange), swimming (20 min; blue) and two alternating dark (10 min each; gray) and light (10 min each; yellow) phases. The average locomotor activity ± the SEM (shaded) per second is shown for zebrafish *AB* wildtype (green; *n* = 60), *crystal* (blue; *n* = 60), and *Dc* (red; *n* = 40) larvae; color-coded arrowheads highlight the increases in locomotor activity 1 s after the illumination switch. **(B,C)** Violin plots of the velocity during movement **(B)** and percentage of time spent moving **(C)** of zebrafish *AB* wildtype, *crystal*, and *Dc* during the habituation (orange) and swimming phase (blue); the mean is indicated by a dotted black line. **(D,E)** Violin plots of the velocity during movement **(D)** and percentage of time spent moving **(E)** of zebrafish *AB*, *crystal*, and *Dc* during the light (yellow) and dark phases (gray); the mean is indicated by a dotted black line. Two-way ANOVA followed by Šídák’s or Tukey’s multiple comparisons test was used to analyze differences in velocity or movement between phases of the light-dark test in and between *AB*, *crystal*, and *Dc*; *p* > 0.05 is abbreviated as not significant (n.s.).

The total duration of the light-dark test is 80 min (4,800 s). In its layout, the test that we used here is identical to the one previously reported by Fitzgerald et al. (2019) and consists of 6 phases: 1 habituation phase (20 min); 1 swimming phase (20 min); 2 dark phases (10 min each); and 2 light phases (10 min each; Figure 1A).

### *Danionella* and *AB* wildtype zebrafish larvae have a similar baseline locomotor activity

During the habituation and swimming phase of the light-dark test, 6 dpf *Dc* and *AB* wildtype zebrafish showed a similar locomotor activity (total distance over time) and velocity during movement (hereafter referred to as velocity; see Section “Materials and methods” for details; Figures 1A,B) *crystal* larvae, however, showed a lower locomotor activity and velocity compared to *AB* zebrafish and *Dc* (Figures 1A,B). In the swimming phase, the average velocity of *Dc* and *AB* zebrafish was 4.24 ± 0.43 and 3.70 ± 0.18 mm/s, respectively, whereas *crystal* larvae moved with a slower average speed of 2.36 ± 0.11 mm/s [Figure 1B; two-way ANOVA followed by Tukey’s multiple comparisons test *F*(2,314) = 30.86, *AB* versus *Dc p* = 0.2406; *AB* versus *crystal p* < 0.0001; *crystal* versus *Dc p* < 0.0001]; we did not observe significant differences in locomotor activity or velocity between the habituation and the swimming phase in *Dc*, *AB*, and *crystal* larvae [two-way ANOVA *F*(1,314) = 0.2000, *p* = 0.6550; Figures 1A,B]. Similarly, we found no differences in the time spent moving between the habituation and swimming phase in zebrafish *AB*, *crystal*, and *Dc* larvae [Figure 1C; two-way ANOVA *F*(1,314) = 0.4462, *p* = 0.5046]. With 58.53 ± 3.81 and 58.25 ± 3.22 percent during the swimming phase, *Dc* and *AB* zebrafish, respectively, were found to spent nearly equal amounts of time moving, whereas with 74.25 ± 2.24 percent *crystal* larvae were found to be moving significantly more [Figure 1C; two-way ANOVA followed by Tukey’s multiple comparisons test *F*(2,314) = 19.00, *AB* versus *Dc p* = 0.9979; *AB* versus *crystal p* = 0.0002; *crystal* versus *Dc p* = 0.0030].

### *Danionella* and zebrafish larvae show similar increases in locomotor activity during dark periods

Similar to *AB* and *crystal* zebrafish, *Dc* strongly increase their locomotor activity and velocity during the dark relative to the light phases [Figures 1A,D; two-way ANOVA followed by Šídák’s multiple comparisons test *F*(1,314) = 297.0, dark versus light phases *AB p* < 0.0001; *crystal p* < 0.0001; *Dc p* < 0.0001]. Compared to the light phases, *AB* showed a 1.4-fold (3.09 ± 0.09 versus 4.39 ± 0.10 mm/s), *crystal* a 2.3-fold (2.53 ± 0.10 versus 5.75 ± 0.17 mm/s), and *Dc* larvae a 1.6-fold (3.96 ± 0.28 versus 6.32 ± 0.26 mm/s) increase in their average velocity during the dark phases (Figure 1D). Thus, the average velocity of *Dc* during both the dark and the light phases was significantly higher than in both *AB* and *crystal* zebrafish [Figure 1D; two-way ANOVA followed by Tukey’s *post hoc* multiple comparison test *F*(2,314) = 35.33 dark phases *AB* versus *Dc p* < 0.0001; *crystal* versus *Dc p* < 0.0451; light phases *AB* versus *Dc p* < 0.0002; *crystal* versus *Dc p* < 0.0001]. In contrast to *AB* and *crystal* zebrafish larvae that increased the percentage of their time spent moving from 53.83 ± 2.70 and 76.04 ± 2.12 during the light to 74.99 ± 1.30 and 91.66 ± 0.74, respectively, during the dark phases, *Dc* spent similar amounts of time moving in the light and dark phases (66.79 ± 2.89 light versus 67.31 ± 3.03 percent dark; Figure 1E).

### *Danionella* and zebrafish larvae show different light-dark and dark-light startle responses

**FIGURE 2:**
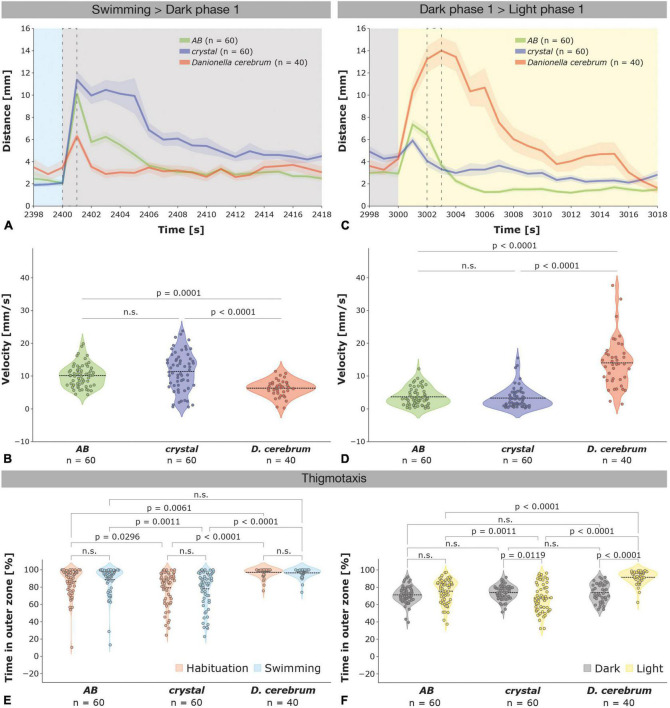
Different startle responses evoked by illumination changes in zebrafish and *Dc* larvae. **(A)** Startle responses ± SEM (shaded) of 6 dpf zebrafish *AB* wildtype (green; *n* = 60), *crystal* (blue; *n* = 60), and *Dc* (red; *n* = 40) larvae depicted from 2 s before (2,398 s) to 18 s after (2,418 s) the first light (swimming phase; blue) to dark (dark phase 1; gray) switch (see also Supplementary Figure 1A); a dotted black rectangle indicates the 1 s time interval that was used to compare the velocity of the larvae in **(B)**. **(B)** Violin plots depicting the velocity of *AB*, *crystal*, and *Dc* larvae during 1 s (2,400–2,401 s) following the first light to dark switch (see also Supplementary Figure 1B). Note that *Dc* increase their velocity significantly less than *AB* wildtype and *crystal* zebrafish. **(C)** Startle responses of 6 dpf zebrafish *AB* wildtype (green), *crystal* (blue), and *Dc* (red) larvae depicted from 2 s before (2,998 s) to 18 s after (3,018 s) the first dark (gray) to light (yellow) switch (see also Supplementary Figure 1C); a dotted black rectangle indicates the 1 s time interval that was used to compare the velocity of the larvae in **(D)**. **(D)** Violin plots depicting the velocity of *AB*, *crystal*, and *Dc* larvae during 1 s (3,002–3,003 s) following the first dark to light switch (see also Supplementary Figure 1D). Note that *Dc* increase their velocity significantly more and during a longer time period than *AB* wildtype and *crystal* zebrafish. One-way ANOVA followed by Tukey’s multiple comparisons test or Kruskal–Wallis test followed by Dunn’s multiple comparisons test was used to analyze differences in velocity between *AB*, *crystal*, and *Dc*; *p* > 0.05 is abbreviated as not significant (n.s.). **(E)** Violin plots depicting the time spent in the outer zone during the habituation (orange) and swimming (blue) phase is highest in *Dc* followed by *AB*, but lower in *crystal* larvae. **(F)** Violin plots show that thigmotaxis is increased in *Dc* relative to both *AB* and *crystal* zebrafish larvae during the light but not the dark phases. Two-way ANOVA followed by Šídák’s or Tukey’s multiple comparisons test was used to analyze differences in thigmotaxis between phases of the dark-light test in and between *AB*, *crystal*, and *Dc*; *p* > 0.05 is abbreviated as not significant (n.s.).

Zebrafish and *Dc* larvae differ most strikingly in their startle behavior immediately after the switch from light to dark and dark to light (Figures 1A, 2A–D and Supplementary Figure 1). *AB* and *crystal* zebrafish strongly increase their locomotor activity immediately after the switch from the swimming to the first dark phase (Figures 1A, 2A) and, similarly, during the switch from the first light to the second dark phase (Figure 1A and Supplementary Figure 1A), and in particular during the first second following the switch (Figure 2A and Supplementary Figure 1A). *Dc* also show a startle response during the first second of the light to dark switch, however, it is much less pronounced than in zebrafish (Figure 2A and Supplementary Figure 1A). With an average velocity of 11.38 ± 0.77 mm/s the amplitude of the startle response was highest in *crystal* followed by 10.14 ± 0.45 mm/s in *AB* but was only 6.32 ± 0.39 mm/s in *Dc* larvae which was significantly different from both *crystal* and *AB* [Figures 1A, 2A,B; one-way ANOVA followed by Tukey’s *post hoc* multiple comparison test *F*(2,157) = 16.53, *AB* versus *Dc p* = 0.0001; *crystal* versus *Dc p* < 0.0001], while there was no difference between *AB* and *crystal* zebrafish larvae [one-way ANOVA followed by Tukey’s *post hoc* multiple comparison test *F*(2,157) = 16.53, *AB* versus *crystal p* = 0.2696]. Differences between zebrafish and *Dc* in the amplitude of their startle response were even more pronounced when switching the illumination from dark to light albeit now with opposing amplitudes relative to the light to dark switch (Figures 1A, 2C,E and Supplementary Figures 2C,E). *Dc* showed the highest amplitude in the startle response 3 s after the switch, whereas wildtype *AB* and *crystal* zebrafish showed the highest response again after 1 s (Figure 2C). Three seconds after the dark to light switch *Dc* peaked with an average velocity of 14.01 ± 1.21 mm/s that was significantly higher compared to the average velocity of 3.69 ± 0.35 and 3.31 ± 0.41 mm/s that we observed in *AB* in *crystal* larvae, respectively (Figures 2C,D; Kruskal–Wallis test followed by Dunn’s multiple comparison test *AB* versus *Dc p* < 0.0001; *crystal* versus *Dc p* < 0.0001; *AB* versus *crystal p* > 0.9999). Moreover, we did not detect a freezing phase, i.e., a decrease in locomotor activity below baseline levels following the dark > light switch in *Dc* although it was manifest in *AB* wildtype and, to a lesser extent, in *crystal* zebrafish larvae (Figures 1A, 2C and Supplementary Figure 1C).

### *Danionella* show increased thigmotaxis relative to zebrafish larvae in the light

Thigmotaxis or centrophobism, i.e., the tendency of animals to avoid the center area of an open field or arena and instead to spend more time in its periphery, is a behavioral response that is evolutionary conserved from *Drosophila* (Besson and Martin, 2005; Mohammad et al., 2016) to zebrafish (Colwill and Creton, 2011; Richendrfer et al., 2012; Schnörr et al., 2012; Pietri et al., 2013; Zhang et al., 2017; Xu and Guo, 2020) and mammals (Hall, 1934; Denenberg, 1969; Treit and Fundytus, 1988; Prut and Belzung, 2003), including humans (Walz et al., 2016; Gromer et al., 2021). Although a natural behavioral tendency across species, it has been suggested that thigmotaxis is indicative of an anxiety-like state in both larval and adult zebrafish (Maximino et al., 2010; Richendrfer et al., 2012; Schnörr et al., 2012; Pietri et al., 2013; Zhang et al., 2017; Abreu et al., 2020; Xu and Guo, 2020).

During the habituation and swimming phase, thigmotaxis was highest in *Dc*, followed by wildtype *AB* zebrafish, whereas *crystal* larvae spent comparatively less time in the outer zone of the wells (Figure 2E); we did not observe significant differences between the habituation and swimming phase in *Danionella* or zebrafish [two-way ANOVA *F*(1,314) = 0.0001574, *p* = 0.9900]. In the swimming phase, *Dc* were found 96.40 ± 0.81 percent of the time in the outer zone while *AB* and *crystal* zebrafish engaged in thigmotactic behavior 88.62 ± 2.16 and 77.95 ± 2.74 percent of the time, respectively. The thigmotactic behavior of *crystal* larvae was therefore significantly lower compared to both *AB* zebrafish and *Dc* whereas the difference between *AB* and *Dc* was not [Figure 2E; two-way ANOVA followed by Tukey’s multiple comparison test *F*(2,314) = 30.45, *crystal* versus *AB p* = 0.0011; *crystal* versus *Dc p* < 0.0001; *AB* versus *Dc p* = 0.0512]. Relative to the light *Dc* decreased thigmotaxis during the dark phases (from 91.58 ± 1.21 in the light to 73.79 ± 1.70 percent in the dark) but showed significantly increased levels of thigmotactic behavior relative to both wildtype *AB* (71.21 ± 1.28 in the dark and 75.55 ± 1.78 percent in the light) and *crystal* zebrafish (73.88 ± 1.07 in the dark and 67.62 ± 2.13 in the light) during the light phases [Figure 2F; two-way ANOVA followed by Tukey’s multiple comparison test *F*(2,314) = 25.69, *Dc* versus *AB p* < 0.0001; *Dc* versus *crystal p* < 0.0001]. Thus, the thigmotactic behavior of *AB* wildtype zebrafish was only mildly altered in the dark versus the light phases, whereas *crystal* and *Dc* larvae responded with an opposing behavior, namely by an increase and a decrease in thigmotaxis, respectively, in the dark relative to the light phases (Figure 2F).

### Age-dependent locomotor activity and light-dark and dark-light startle responses in 4–6 dpf *Dc* and *AB* zebrafish larvae

**FIGURE 3:**
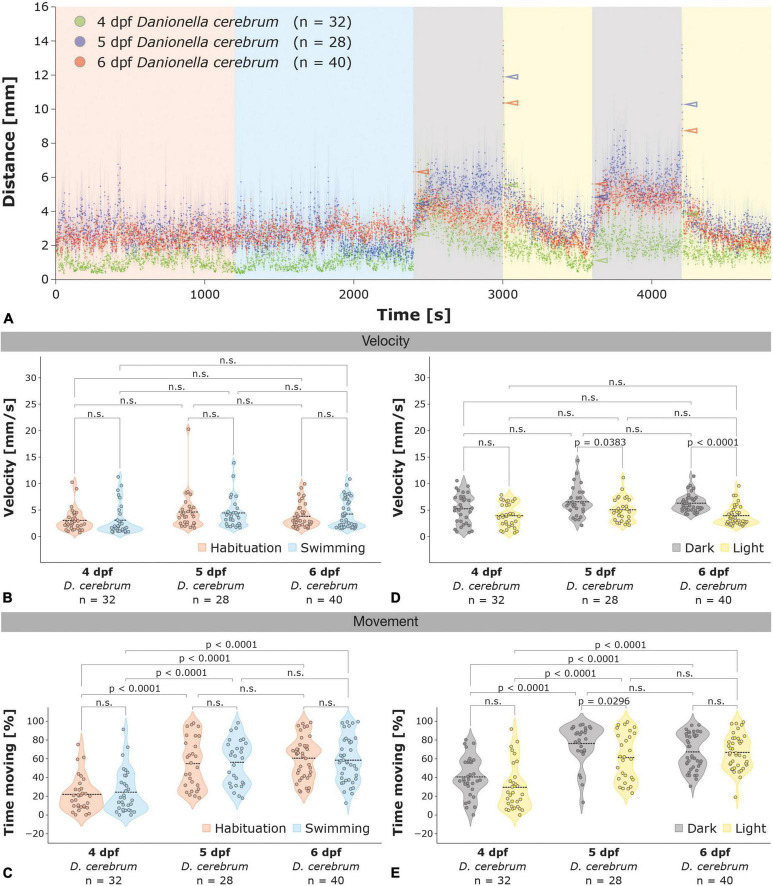
Locomotor activity of 4–6 dpf *Dc* larvae in the light-dark test. **(A)** Locomotor activity of 4 dpf (green; *n* = 32), 5 dpf (blue; *n* = 28), and 6 dpf (red; *n* = 40) *Dc* larvae in the light-dark test; color-coded arrowheads highlight the increases in locomotor activity 1 s after the illumination switch. **(B,C)** Violin plots of the velocity during movement **(B)** and the time spent moving **(C)** for 4–6 dpf *Dc* larvae in the habituation (red) and swimming (blue) phase. **(D,E)** Violin plots of the velocity during movement **(D)** and the time spent moving **(E)** for 4–6 dpf *Dc* larvae in the light (yellow) and dark (gray) phases. Note the reduced movement of 4 dpf **(C,E)** and the lack of increase in velocity during the dark phases **(A,D)** relative to 5 and 6 dpf *Dc* larvae. Two-way ANOVA followed by Šídák’s or Tukey’s multiple comparisons test was used to analyze differences in velocity or movement between phases of the light-dark test in and between 4 and 6 dpf *Dc*; *p* > 0.05 is abbreviated as not significant (n.s.).

How does the locomotor activity of *Danionella* larvae change during development and at what developmental age do larvae respond to changes in illumination in the light-dark test? To answer these questions we investigated the locomotor activity in 4 and 5 dpf *Dc* and compared it to 6 dpf larvae. We found that the velocity during movement in the swimming phase was lower in 4 dpf *Dc* (3.09 ± 0.51 mm/s) compared to 5 dpf (4.45 ± 0.55 mm/s) and 6 dpf (4.24 ± 0.43 mm/s) larvae, although this difference was not significant [Figures 3A,B; two-way ANOVA followed by Šídák’s multiple comparisons test *F*(2,194) = 4.563, 4 dpf versus 5 dpf *p* = 0.1583; 4 dpf versus 6 dpf *p* = 0.2180]. However, the percentage of time during the swimming phase that 4 dpf (24.33 ± 4.02%) spent moving was less than half that of 5 dpf (56.21 ± 4.53%) and 6 dpf (58.53 ± 3.81%) larvae [Figure 3C; two-way ANOVA followed by Tukey’s multiple comparisons test *F*(2,194) = 49.85, 4 dpf versus 5 dpf *p* < 0.0001; 4 dpf versus 6 dpf *p* < 0.0001] whereas there was no difference between 5 dpf and 6 dpf [Figure 3C; two-way ANOVA followed by Tukey’s multiple comparisons test *F*(2,194) = 49.85, 5 dpf versus 6 dpf *p* = 0.9130]. Increases in the velocity during the dark relative to the light phases were found significant in 5 and 6 dpf *Dc* larvae whereas in 4 dpf they were not [Figure 3D; two-way ANOVA followed by Šídák’s multiple comparisons test *F*(1,194) = 28.95, 6 dpf *p* < 0.0001; 5 dpf *p* = 0.0383; 4 dpf *p* = 0.0600]. Similar to the habituation and swimming phases, 5 and 6 dpf generally spent more time moving than 4 dpf during both the dark and the light phases, and 4 and 5 dpf tended to spend a higher percentage of time moving during the dark relative to the light phases whereas 6 dpf *Dc* larvae did not (Figure 3E).

**FIGURE 4:**
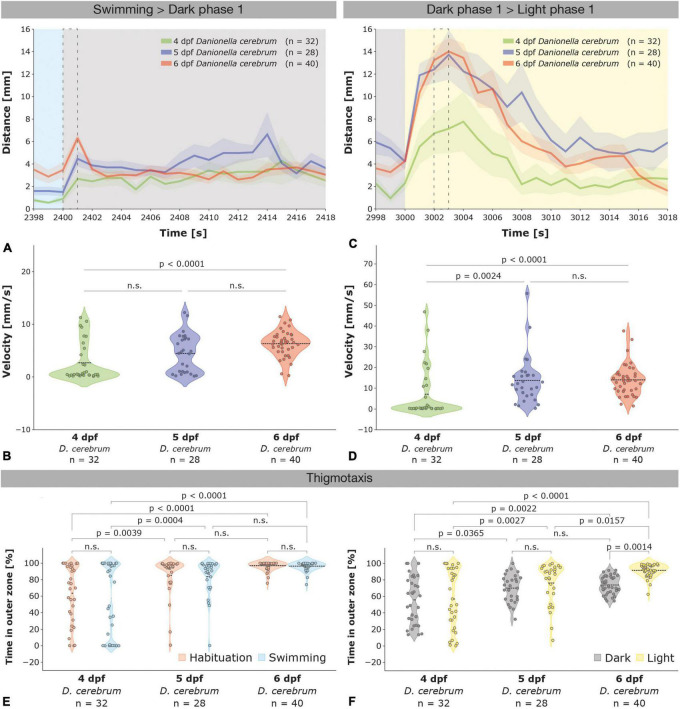
Age-dependent startle responses evoked by illumination changes and thigmotaxis in 4–6 dpf *Dc* larvae. **(A)** Startle responses with standard error of the mean (SEM; shaded area) of 4 dpf (green, *n* = 32), 5 dpf (blue, *n* = 28), and 6 dpf (red, *n* = 40) *Dc* larvae depicted from 2 s before (2,398 s) to 18 s after (2,418 s) the first light (swimming phase; blue) to dark (dark phase 1; gray) switch (see also Supplementary Figure 2A); a dotted black rectangle indicates the 1 s time interval that was used to compare the velocity of the larvae in **(B)**. **(B)** Violin plots depicting the velocity of 4–6 dpf *Dc* larvae during 1 s (2,400–2,401 s) following the first light to dark switch (see also Supplementary Figure 2B). **(C)** Startle responses of 4–6 dpf *Dc* larvae depicted 2 s before (2,998 s) and 18 s after (3,018 s) the first dark (gray) to light (yellow) switch; see also Supplementary Figure 2C; a dotted black rectangle indicates the 1 s time interval that was used to compare the velocity of the larvae in **(D)**. **(D)** Violin plots depicting the velocity of 4–6 dpf *Dc* larvae during 1 s (3,002–3,003 s) following the first dark to light switch (see also Supplementary Figure 2D). Note that 5 and 6 dpf *Dc* larvae increase their velocity significantly more than 4 dpf. Kruskal–Wallis test followed by Dunn’s multiple comparisons test was used to analyze differences in velocity between 4 and 6 dpf *Dc*; *p* > 0.05 is abbreviated as not significant (n.s.). **(E,F)** Violin plots depicting the time spent in the outer zone of the wells show an age-dependent increase in thigmotaxis in 5 and in 6 dpf compared to 4 dpf *Dc* larvae during the habituation and swimming **(E)** and also during the light and dark phases **(F)**. Two-way ANOVA followed by Šídák’s or Tukey’s multiple comparisons test was used to analyze differences in thigmotaxis between phases of the light-dark test in and between 4 and 6 dpf *Dc*; *p* > 0.05 is abbreviated as not significant (n.s.).

During the light to dark and dark to light switches, 4 dpf *Dc* exhibited a startle response, however, it was overall less pronounced than in 5 and 6 dpf larvae that showed comparatively similar responses (Figures 4A–D and Supplementary Figure 2). One second after the first light to dark switch 4, 5, and 6 dpf had an average velocity of 2.68 ± 0.65 mm/s, 4.46 ± 0.69 mm/s, and 6.32 ± 0.39 mm/s, respectively (Figures 4A,B; Kruskal–Wallis test followed by Dunn’s multiple comparisons test 4 dpf versus 5 dpf *p* = 0.0865; 4 versus 6 dpf *p* < 0.0001; 5 versus 6 dpf *p* = 0.1678). As previously shown for 6 dpf, the amplitude of the startle response of 4 and 5 dpf *Dc* larvae in a dark to light switch was 2–3 times higher relative to a light to dark switch peaking at an average of 7.14 ± 2.16 mm/s in 4 dpf, 13.72 ± 2.21 mm/s in 5 dpf, and 14.01 ± 1.21 mm/s in 6 dpf after 3 s following the switch (Figures 4C,D; Kruskal–Wallis test followed by Dunn’s multiple comparisons test 4 dpf versus 5 dpf *p* = 0.0024; 4 versus 6 dpf *p* < 0.0001; 5 versus 6 dpf *p* > 0.9999).

The described development of locomotor activity in 4–6 dpf *Dc* is largely similar to the development of locomotor activity in 4–6 dpf *AB* zebrafish larvae. Four dpf *AB* also show a lower locomotor activity (Supplementary Figure 3A) and velocity (Supplementary Figures 3B,D) compared to 5 and 6 dpf *AB* larvae throughout all phases of the dark-light test. Although the time spent moving of 4 dpf *AB* during the swimming phase (37.77 ± 3.67%) is not less than half of that of 5 (72.68 ± 3.14%) and 6 dpf (58.29 ± 3.22%) *AB* larvae, as it is the case in 4 versus 5 and 6 dpf *Dc* (see above), it is still substantially reduced during all but the dark phases (Supplementary Figures 3C,E). The overall similarities in locomotor activity of 5 and 6 dpf *AB*, but not 4 dpf *AB* zebrafish, further extend to similarities in their corresponding startle responses during the light to dark and dark to light switches (Supplementary Figures 4A–D, 5A–D). Here, we found the increases in the velocity during the first second following the first (Supplementary Figures 4A,B) and second (Supplementary Figures 5A,B) light to dark switch of 5 and 6 dpf *AB* zebrafish to be significantly different from 4 dpf but quite similar in 5 and 6 dpf larvae (Supplementary Figure 4B; Kruskal–Wallis test followed by Dunn’s multiple comparisons test 4 dpf versus 5 dpf *p* = 0.0182; 4 versus 6 dpf *p* = 0.0339; 5 versus 6 dpf *p* > 0.9999). More pronounced than 4 dpf but similar in between 5 and 6 dpf *AB* zebrafish startle responses were also seen in both dark to light switches (Supplementary Figures 4C,D, 5C,D). However, whereas the startle response of 6 dpf *AB* larvae exhibited a significantly higher amplitude relative to 4 dpf *AB* during both dark to light switches, the increase in velocity of 5 dpf relative to 4 dpf reached significance only during the second (Supplementary Figure 5D) but not during the first (Supplementary Figure 4D) dark to light switch.

### Age-dependent thigmotaxis of 4–6 dpf *Danionella* versus age-independent thigmotaxis of zebrafish larvae

During all (habituation, swimming, dark, and light) phases of the light-dark test, thigmotaxis was higher in 5 and 6 dpf *Danionella* compared to 4 dpf larvae (Figures 4E,F). During the swimming phase, for example, 4 dpf larvae spent only slightly more than half of their time (57.34 ± 7.74%) in the outer zone of the wells whereas 5 dpf (83.54 ± 4.23%) and 6 dpf (96.40 ± 0.81%) larvae spent most of their time in the outer zone [Figure 4E; two-way ANOVA followed by Tukey’s multiple comparisons test *F*(2,194) = 35.37, 4 dpf versus 5 dpf *p* = 0.0004; 4 dpf versus 6 dpf *p* < 0.0001; 5 dpf versus 6 dpf *p* = 0.1107]. Thigmotaxis decreased during the dark phases relative to light phases in all developmental ages of *Dc*, although this effect was most strongly seen at 6 dpf [Figures 4F; 4 dpf: 56.84 ± 6.36% light phases versus 55.72 ± 5.03% dark phases; 5 dpf: 76.23 ± 4.71% light phases versus 70.06 ± 3.16% dark phases; 6dpf: 91.58 ± 1.21% versus 73.80 ± 1.70% dark phases; two-way ANOVA followed by Šídák’s multiple comparisons test *F*(1,194) = 6.886, 4 dpf *p* = 0.9960; 5 dpf *p* = 0.6584; 6 dpf *p* = 0.0014]. Thus, *Dc* exhibit an age-dependent thigmotaxis that increases from 4 to 6 dpf and decreases during the dark relative to light periods. This age-dependent thigmotaxis of *Dc* is distinct from an age-independent thigmotaxis of *AB* zebrafish, however, as we found the percentage of time spent in the outer zone of the wells throughout all phases of the light-dark test largely unchanged in 4–6 dpf *AB* larvae (compare Figures 4E,F for 4–6 dpf *Dc* with Supplementary Figures 4E,F for 4–6 dpf *AB* zebrafish). Analogous to 4–6 dpf *Dc* though, 4–6 dpf *AB* zebrafish also exhibited a reduced thigmotaxis during the dark relative to the light periods (compare Figure 4F with Supplementary Figure 4F).

## Discussion

Making use of a light-dark test, we analyzed and compared the larval locomotor activity of *AB* wildtype and *crystal* zebrafish with *D. cerebrum*, an emerging neurophysiological model species (Britz et al., 2021; Rajan et al., 2022a). Furthermore, we compared and analyzed the development of larval locomotor activity in 4, 5, and 6 dpf *Dc* and *AB* zebrafish.

### Development of locomotor activity and thigmotaxis in *Dc* compared to zebrafish larvae

Overall, the ontogenetic development of larval locomotor activity in 4–6 dpf *Dc* and *AB* zebrafish appears to be largely similar. In *Dc* as well as in *AB* zebrafish, 4 dpf show a lower locomotor activity and spend more time resting compared to 5 and 6 dpf larvae; for zebrafish this has also been previously described by Colwill and Creton (2011), Padilla et al. (2011), and Ingebretson and Masino (2013). Both 4–6 dpf *Dc* and *AB* zebrafish decrease their resting time and increase their velocity in the dark relative to the light phases. *Dc* and *AB* zebrafish also both exhibit an age-dependent response manifest in how the two species respond to changes in illumination. Here, 5 and 6 dpf *Dc* as well as 5 and 6 dpf *AB* zebrafish show a comparable amplitude in their startle responses that is different and less pronounced at 4 dpf in both species. In 4 dpf *Dc* we occasionally observed Rosetta-like locomotor activity patterns of concentric trajectories (Supplementary Figures 6A–C); such activity patterns, however, were not observed in zebrafish larvae (Supplementary Figure 6D).

Due to their lack of pigmentation, reduced movement, and optical translucency we were unable to reliably detect and track 4 dpf *crystal* larvae with our system. In general, tracking and, in particular, detection of non-moving *crystal* larvae proved to be challenging and more difficult than detection and tracking of *AB* zebrafish or *Dc*. Even at 5 dpf *crystal* larvae were moving less than half of the time (<50%) during all test phases except the dark phase, possibly indicating a slightly delayed development compared to *AB* wildtype, whereas 6 dpf *crystal* moved significantly more (>74%; Supplementary Figures 7C,E). Aside from differences in movement, 5 and 6 dpf *crystal* larvae showed an overall similar locomotor activity (Supplementary Figure 7A), velocity (Supplementary Figures 7B,D), and responses to changes in illumination (Supplementary Figures 7A, 8A–D, 9A–D). However, the amplitude of the startle response following the light to dark (Supplementary Figures 8A, 9A) and dark to light switch (Supplementary Figures 8A, 9A) appeared to be more variable and less consistent in 5 dpf in relation to 6 dpf *crystal* larvae, suggesting comparable behavioral analyses are best performed at 6 dpf (Fitzgerald et al., 2019).

In contrast to 6 dpf *AB* and *Dc* that decreased thigmotaxis during the dark relative to the light phases, 6 dpf *crystal* zebrafish apparently increased their time spent in the outer zone of the wells during the dark (Figure 2F); we did not observe increased thigmotaxis during the dark in 5 dpf *crystal* larvae though (Supplementary Figure 8F). The transparent nature due to the lack of pigmentation of *crystal* larvae could possibly bias them to increase thigmotaxis in the dark as a predator avoidance behavior. However, as increased thigmotaxis during the dark was not observed in 5 dpf *crystal*, it is also possible that thigmotaxis is generally more variable in *crystal* larvae and that individuals of this pigmentation mutant exhibit a higher individual variability compared to *AB* zebrafish.

The age-dependent thigmotaxis in 4–6 dpf *Dc* contrasts with age-independent thigmotaxis that we and others (e.g., Colwill and Creton, 2011) observed for *AB* zebrafish of the same age. The Rosetta-like locomotor activity pattern, in which 4 dpf *Dc* swim in concentric trajectories mostly within the center of the wells and which is absent in their evolutionary closely related zebrafish counterparts and also rarely seen in *Dc* older than 4 dpf, may contribute to this apparent age-dependency; however, since we observe this pattern only occasionally and not in all individuals, it may be a contributing but not a determining factor. In principle, an increase in movement, as it is seen from 4 to 5 and 6 dpf in *Dc* (Figures 3C,E), coupled with an increase in the ratio of straight paths versus turns could also lead itself to an increase in thigmotaxis when locomotor activity is measured in arenas with concave walls, as has been pointed out by Fero et al. (2010) and Horstick et al. (2016). However, considering the fact that 4 dpf *AB* zebrafish also show a largely reduced amount of time spent moving compared to their 5 and 6 dpf counterparts (Supplementary Figures 3C,E), but, at the same time, exhibit no major differences in the time spent in the outer zone of the wells (Supplementary Figures 4E,F), makes an explanation relying solely on an increase of straight forward motion at the expenditure of turns rather unlikely, even though we are currently lacking information about how such a glide and turn ratio compares between *Dc* and zebrafish.

That *Dc* larvae show an increased thigmotaxis relative to zebrafish during the light (Figure 2F), together with a strong startle response during a dark to light switch (Figures 2C,D and Supplementary Figures 1C,D), and the observation that *Dc* preferentially occupy the lower zone of a water column (Rajan et al., 2022b, see also below) appears to be indicating that *Dc* may generally favor a rather dark over a light environment. Since thigmotaxis has also been associated with an anxiety-like behavior (Maximino et al., 2010; Richendrfer et al., 2012; Schnörr et al., 2012; Pietri et al., 2013; Zhang et al., 2017; Abreu et al., 2020; Xu and Guo, 2020), increased thigmotaxis of *Dc* relative to zebrafish during the light periods could also be indicating increased levels of anxiety in *Dc*. Although we cannot exclude this possibility, ascribing heightened levels of anxiety to *Dc* compared to zebrafish based solely on a single behavioral parameter appears to be premature, which is why we currently favor a natural habitat or environmental-based hypothesis as a more plausible explanation for the observed phenomena.

### Different natural habitats may be underlying different startle responses in *Dc* and zebrafish larvae

Although the baseline locomotor activity is comparatively similar in 6 dpf wildtype *AB* zebrafish and *Dc*, but not *crystal*, and both species increase their velocity during the dark relative to the light phases, they differ strikingly in their startle response to sudden changes in illumination (Figures 1A, 2A–D and Supplementary Figure 1). Whereas *AB* and *crystal* larvae respond strongly to a light > dark switch but only weakly to a dark > light switch, Dc respond strongly to a dark > light switch and only weakly to a light > dark switch. What may be causing this differential response in the two evolutionary closely related species? One possible explanation may be that *D. rerio* and *D. cerebrum* occupy different depths within the water column of the slow flowing streams, pools, and ponds of northeastern India and Myanmar that form their natural habitat and where they may or may not sometimes even co-exist. Indeed, it has been reported that *D. cerebrum* was found at a depth below 30 cm of the water surface (Britz et al., 2021) and with adults spawning in crevices and small openings at the bottom of laboratory tanks (Schulze et al., 2018) which is in contrast to zebrafish that spawn in shallow and typically clear water near the surface (Parichy, 2015). Reports from observations of the two species in their natural habitats were recently further confirmed in the laboratory by directly showing that 6 dpf *Dc* predominantly (∼80%) occupy the lower zone (0–12 cm) whereas 6 dpf zebrafish larvae predominantly (∼80%) occupy the upper zone (24–36 cm) of a water column with a total height of 36 cm (Rajan et al., 2022b). Larval zebrafish may thus generally be more accustomed to a brighter environment thereby triggering a strong light > dark but a comparatively weaker dark > light startle response, whereas larval *Dc* may generally be more accustomed to a darker environment thereby triggering a strong dark > light but a comparatively weaker light > dark startle response. Since both zebrafish, in particular in the *crystal* background (Antinucci and Hindges, 2016; Mattern et al., 2020), and *Dc* are uniquely amenable to whole-brain *in vivo* imaging techniques (Ahrens et al., 2013; Schulze et al., 2018; Rajan et al., 2022b) the different light-dark and dark-light response in both species may possibly represent an interesting opportunity for a comparative neurophysiological analysis of the mechanisms and evolution of neural circuits in two closely related vertebrate species through which they evoke different behavioral responses to similar environmental stimuli.

## Materials and methods

### Zebrafish and *Danionella* maintenance

Zebrafish (*D. rerio*) and *D. cerebrum* were maintained and raised at 28°C on a 14 h light/10 h dark cycle and bred following standard procedures (Schulze et al., 2018; Aleström et al., 2019; Rajan et al., 2022a). *Danionella* eggs were collected from spontaneous spawnings, and both species were raised in 30% Danieau solution [17.4 mM NaCl, 0.21 mM KCl, 0.12 mM MgSO_4_, 0.18 mM Ca(NO_3_)_2_, 1.5 mM HEPES, pH 7.0] in 94 mm (diameter) × 16 mm (depth) petri dishes (Greiner Bio-One, Kremsmünster, Austria).

### Experimental setup

The experimental setup consisted of custom-made black box with the dimensions 666 mm (length) × 472 mm (width) × 1010 mm (height) fabricated by Noldus (Wageningen, Netherlands) that shielded larvae from external influences. The box was illuminated through light emitting diodes (LEDs) of a white light and infrared (IR; 940–950 nm) backlight unit located at the bottom and contained a Gigabit Ethernet camera (acA1300-60gm; Basler, Ahrensburg, Germany) attached to 12 mm/F1.4 lens (Kowa, Nagoya, Japan) with an 850 nm IR filter (Heliopan, Gräfelfing, Germany) on the top. The white light unit was connected to an USB-IO box Noldus (Wageningen, Netherlands) that was controlled through EthoVision XT software (15.0.1418, Noldus, Wageningen, Netherlands) running under Windows 10 Pro (Microsoft, Redmond, WA, United States) on a Dell (Round Rock, TX, United States) workstation. 12-well plates (Greiner Bio-One, Kremsmünster, Austria) filled with 4 ml of Danieau solution were placed directly on top of the IR (940 nm) and white light illumination unit. Illuminance inside the wells was measured at 1,300 lux with a Panlux electronic 2 photometer [Gossen Metrawatt (previously Gossen) Nürnberg, Germany]. A Fresnel lens (Noldus, Wageningen, Netherlands) was placed in between the Gigabit Ethernet camera and the 12-well plate (at a distance of 20 and 265 mm from the 12-well plate and the Gigabit Ethernet camera, respectively) to reduce distortion of non-centered wells relative to the camera’s position and to increase the contrast within and in particular at the border of the wells in order to optimize IR tracking quality and robustness. The temperature of the room that contained the experimental setup was maintained at 28°C.

### Light-dark test and tracking

The light dark test was performed as previously reported by Fitzgerald et al. (2019) with slight modifications. 24 h before the start of the behavioral analysis larvae were transferred from a 94 mm (diameter) × 16 mm (depth) petri dish (Greiner Bio-One, Kremsmünster, Austria) into individual wells [22.2 mm (diameter) × 16.5 mm (depth)] filled with 4 ml Danieau solution on a 12-well plate (Greiner Bio-One, Kremsmünster, Austria) to accustom to the new environment. On the day of the experiment, larvae in the 12-well plates were transferred from the incubator in which they were raised to the experimental setup at 12:00 p.m. to which they were allowed to accustom for 1 h before the light-dark test was started at 1:00 p.m. The light-dark test was thus always carried out at the same time of the day and experimental parameters were kept constant to avoid as much as possible potential effects on locomotor activity as has been reported previously (MacPhail et al., 2008; Ingebretson and Masino, 2013).

Live video tracking was performed with 30 frames per second (fps) at a resolution of 1,280 pixels × 1,024 pixels with EthoVision XT software (15.0.1418, Noldus, Wageningen, Netherlands) that also controlled the light-dark and dark-light illumination switches through an USB-IO box (Noldus, Wageningen, Netherlands) that was connected to the custom-made black box containing the white light and IR illumination unit (Noldus, Wageningen, Netherlands). The total duration of the light-dark test was 4,800 s (80 min) and it was divided into the following phases: 0 – 1,200 s habituation phase; 1,201 – 2,400 s swimming phase; 2,401 – 3,000 s dark phase 1; 3,001 – 3,600 s light phase 1; 3,601 – 4,200 s dark phase 2; and 4,201 – 4,800 s light phase 2. The light was switched off at the end of the swimming phase at 2,400 and light phase 1 at 3,600 s; the light was switched on at the end of dark phase 1 at 3,000 and dark phase 2 at 4,200 s.

To measure thigmotaxis of *Danionella* and zebrafish larvae in the light-dark test we defined an outer and an inner zone within each well of the 12-well plate with an equal surface area with EthoVision XT software (15.0.1418, Noldus, Wageningen, Netherlands).

### Data analysis and processing

Data were analyzed and processed with EthoVision XT software (15.0.1418, Noldus, Wageningen, Netherlands) and exported to Microsoft Excel (Microsoft, Redmond, WA, United States). Graphs were generated with plotly in Python^1^ and assembled with Adobe Illustrator (24.3, San Jose, CA, United States). For the analysis of locomotor activity (Figures 1A, 2A,C, 3A, 4A,C and Supplementary Figures 1A,C, 2A,C) the average total distance of *AB*, *crystal* and *Danionella* larvae per second time interval recorded with 30 fps was first exported from EthoVision XT to Microsoft Excel and organized into data sheets. The standard error of the mean (SEM) was then calculated in Python for each time point per second, and the data were visualized with plotly. Similarly, data for velocity (Figures 1B,D, 3B,D), movement (Figures 1C,E, 3C,E), and thigmotaxis (Figures 2E,F, 4E,F) during the swimming, habituation, both light and both dark phases of the light-dark test was also exported from EthoVision XT to Microsoft Excel and visualized with plotly.

A threshold setting of 0.84 and 0.42 mm/s with an averaging interval of 3 frames (100 ms) was defined with EthoVision XT for moving versus non-moving larvae, respectively. This threshold was defined based on the average larval body length of 4.2 mm (and our observations of resting versus moving larvae) that we measured in *AB*, *crystal*, and *Danionella* at 6 dpf (Supplementary Figure 10; see also the section body length measurements below). Thus, larvae moving less than 1/10 of their body length per second were considered not-moving whereas larvae moving more than 1/5 per second were considered moving. Based on this definition we analyzed the velocity in moving larvae to which we also applied an averaging interval across 3 frames (100 ms); no averaging interval, however, was applied for the analysis of the velocity during the 1 s time intervals of the startle responses (Figures 2B,D, 4B,D and Supplementary Figures 1B,D, 2B,D).

### Body length measurements

Body (snout to tail including the fin) and standard length (snout to tail excluding the fin; Parichy et al., 2009) were measured in 6 dpf *AB* wildtype and *crystal* zebrafish and in 4–6 dpf *Danionella* larvae after the light-dark test (Supplementary Figure 10). Single larvae were anesthetized with MS-222 [Merck (previously Sigma-Aldrich) Darmstadt, Germany] in the wells of the 12-well plate and imaged with a Leica M205 FA stereomicroscope (Leica Microsystems, Wetzlar, Germany) controlled by LAS X software (3.4.2.18368; Leica Microsystems, Wetzlar, Germany). Body and standard length were measured with the scale bar tool of LAS X on the acquired images.

### Statistics

Statistical analysis was performed with Prism (9.1.2, GraphPad, La Jolla, CA, United States). A D’Agostino and Pearson and an Anderson-Darling test was used to determine whether the data followed a Normal (Gaussian) distribution. Parametric statistical analysis was performed by one-way (Figures 2B, 4B,D and Supplementary Figures 1B,D, 2B,D, 5B,D, 10) or two-way analysis of variance (ANOVA) (Figures 1B–E, 2E,F, 3B–E, 4E,F and Supplementary Figures 3B–E, 4E,F, 7B–E, 8E,F) followed by Tukey’s or Šídák’s multiple comparisons test as appropriate or an unpaired Student’s *t*-test; (Supplementary Figure 8B); non-parametric statistical analysis was performed by a Kruskal–Wallis test (Figures 2D, 4B,D and Supplementary Figures 2B,D, 4B,D) followed by Dunn’s multiple comparisons test or a Mann–Whitney *U* test (Supplementary Figures 8B,D). *P*-values are shown in the graphs for all values with *p* < 0.05 that was considered significant; *p*-values with *p* > 0.05 were considered as not significant and are abbreviated in the graphs as n.s.

## Data availability statement

The original contributions presented in the study are included in the article/Supplementary material, further inquiries can be directed to the corresponding author.

## Ethics statement

All animal procedures and experiments were conducted in accordance with the European Union Directive 2010/63/EU to reduce and minimize animal suffering and were reviewed and approved by the Lower Saxony State Office for Consumer Protection and Food Safety (33.19-42502-04-21/3827).

## Author contributions

NL, LK, JP, RS, and TK performed the light-dark test under the supervision of JT and contributed to data analysis. JT conceived the project, analyzed the data, and wrote the manuscript together with RK. All authors contributed to the article and approved the submitted version.

## Footnotes

1
https://plotly.com/python/

## Supplementary material

Supplementary Figure 1 (Related to Figures 2A–D). Different startle responses evoked by illumination changes in zebrafish and *Dc* larvae. **(A)** Startle responses ± SEM (shaded) of 6 dpf zebrafish *AB* wildtype (green; *n* = 60), *crystal* (blue; *n* = 60), and *Dc* (red; *n* = 40) larvae depicted from 2 s before (3,598 s) to 18 s after (3,618 s) the second light (yellow) to dark (gray) switch; a dotted black rectangle indicates the 1 s time interval that was used to compare the velocity of the larvae in **(B)**. Note the similar responses of *AB*, *crystal*, and *Dc* larvae compared to the first light to dark switch depicted in Figure 2A. **(B)** Violin plots depicting the velocity of *AB*, *crystal*, and *Dc* larvae during 1 s (3,600–3,601 s) following the second light to dark switch (see also Figure 2B). **(C)** Startle responses of 6 dpf zebrafish *AB* wildtype (green), *crystal* (blue), and *Dc* (red) larvae depicted from 2 s before (4,198 s) to 18 s after (4,218 s) the second dark (gray) to light (yellow) switch; a dotted black rectangle indicates the 1 s time interval that was used to compare the velocity of the larvae in **(D)**. Note the similar responses of *AB*, *crystal*, and *Dc* larvae compared to the first dark to light switch depicted in Figure 2C. **(D)** Violin plots depicting the velocity of *AB*, *crystal*, and *Dc* larvae during 1 s (4,202–4,203 s) following the second dark to light switch (see also Figure 2D). One-way ANOVA followed by Tukey’s multiple comparisons test or Kruskal–Wallis test followed by Dunn’s multiple comparisons test was used to analyze differences in velocity between *AB*, *crystal*, and *Dc*; *p* > 0.05 is abbreviated as not significant (n.s.).

Supplementary Figure 2 (Related to Figures 4A–D). Age-dependent startle responses evoked by illumination changes in 4–6 dpf *Dc* larvae. **(A)** Startle responses with standard error of the mean (SEM; shaded area) of 4 dpf (green, *n* = 32), 5 dpf (blue, *n* = 28), and 6 dpf (red, *n* = 40) *Dc* larvae depicted from 2 s before (3,598 s) to 18 s after (3,618 s) the second light to dark switch (compare with Figure 4A); a dotted black rectangle indicates the 1 s time interval that was used to compare the velocity of the larvae in **(B)**. **(B)** Violin plots depicting the velocity of 4–6 dpf *Dc* larvae during 1 s (3,600–3,601 s) following the second light to dark switch (compare with Figure 4B). **(C)** Startle responses of 4–6 dpf *Dc* larvae depicted 2 s before (4,198 s) and 18 s after (4,218 s) the second dark (gray) to light (yellow) switch; a dotted black rectangle indicates the 1 s time interval that was used to compare the velocity of the larvae in **(D)**. **(D)** Violin plots depicting the velocity of 4–6 dpf *Dc* larvae during 1 s (4,202–4,203 s) following the second dark to light switch. Kruskal–Wallis test followed by Dunn’s multiple comparisons test was used to analyze differences in velocity between 4 and 6 dpf *Dc*; *p* > 0.05 is abbreviated as not significant (n.s.).

Supplementary Figure 3 Locomotor activity of 4–6 dpf *AB* larvae in the light-dark test. **(A)** Locomotor activity of 4 dpf (green; *n* = 48), 5 dpf (blue; *n* = 48), and 6 dpf (red; *n* = 60) *AB* larvae in the light-dark test; color-coded arrowheads highlight the increases in locomotor activity 1 s after the illumination switch. **(B,C)** Violin plots of the velocity during movement **(B)** and the time spent moving **(C)** for 4–6 dpf *AB* larvae in the habituation (red) and swimming (blue) phase. **(D,E)** Violin plots of the velocity during movement **(D)** and the time spent moving **(E)** for 4–6 dpf *AB* larvae in the light (yellow) and dark (gray) phases. Note the reduced velocity **(B,D)** and time spent moving **(C,E)** of 4 dpf particularly during the habituation, swimming and light phases relative to 5 and 6 dpf *AB* larvae. Two-way ANOVA followed by Šídák’s or Tukey’s multiple comparisons test was used to analyze differences in velocity or movement between phases of the light-dark test in and between 4 and 6 dpf *AB*; *p* > 0.05 is abbreviated as not significant (n.s.).

Supplementary Figure 4 Age-dependent startle responses evoked by illumination changes and age-independent thigmotaxis in 4–6 dpf *AB* larvae. **(A)** Startle responses with standard error of the mean (SEM; shaded area) of 4 dpf (green, *n* = 48), 5 dpf (blue, *n* = 48), and 6 dpf (red, *n* = 60) *AB* larvae depicted from 2 s before (2,398 s) to 18 s after (2,418 s) the first light to dark switch (see also Supplementary Figure 5A); a dotted black rectangle indicates the 1 s time interval that was used to compare the velocity of the larvae in **(B)**. **(B)** Violin plots depicting the velocity of 4–6 dpf *AB* larvae during 1 s (2,400–2,401 s) following the first light to dark switch (see also Supplementary Figure 5B). **(C)** Startle responses of 4–6 dpf *AB* larvae depicted 2 s before (2,998 s) and 18 s after (3,018 s) the first dark (gray) to light (yellow) switch; (see also Supplementary Figure 5C); a dotted black rectangle indicates the 1 s time interval that was used to compare the velocity of the larvae in **(D)**. **(D)** Violin plots depicting the velocity of 4–6 dpf *AB* larvae during 1 s (3,000–3,001 s) following the first dark to light switch (see also Supplementary Figure 5D). Note the relatively similar startle responses of 5 and 6 dpf *AB* larvae during the illumination changes. Kruskal–Wallis test followed by Dunn’s multiple comparisons test was used to analyze differences in velocity between 4 and 6 dpf *AB*; *p* > 0.05 is abbreviated as not significant (n.s.). **(E,F)** Violin plots depicting the time spent in the outer zone of the wells show age-independent levels of thigmotaxis in 4–6 dpf *AB* throughout all phases of the test and a significant decrease during the dark phases in 4 dpf **(E)**. Two-way ANOVA followed by Šídák’s or Tukey’s multiple comparisons test was used to analyze differences in thigmotaxis between phases of the light-dark test in and between 4 and 6 dpf *AB*; *p* > 0.05 is abbreviated as not significant (n.s.).

Supplementary Figure 5 (Related to Supplementary Figures 4A–D). Age-dependent startle responses evoked by illumination changes in 4–6 dpf *AB* larvae. **(A)** Startle responses with standard error of the mean (SEM; shaded area) of 4 dpf (green, *n* = 48), 5 dpf (blue, *n* = 48), and 6 dpf (red, *n* = 60) *AB* larvae depicted from 2 s before (3,598 s) to 18 s after (3,618 s) the second light to dark switch (compare with Supplementary Figure 4A); a dotted black rectangle indicates the 1 s time interval that was used to compare the velocity of the larvae in **(B)**. **(B)** Violin plots depicting the velocity of 4–6 dpf *AB* larvae during 1 s (3,600–3,601 s) following the second light to dark switch (compare with Supplementary Figure 4B). **(C)** Startle responses of 4–6 dpf *AB* larvae depicted 2 s before (4,198 s) and 18 s after (4,218 s) the second dark (gray) to light (yellow) switch; a dotted black rectangle indicates the 1 s time interval that was used to compare the velocity of the larvae in **(D)**. **(D)** Violin plots depicting the velocity of 4–6 dpf *AB* larvae during 1 s (4,200–4,201 s) following the second dark to light switch (see also Supplementary Figure 4D). Kruskal–Wallis test followed by Dunn’s multiple comparisons test was used to analyze differences in velocity between 4 and 6 dpf *AB*; *p* > 0.05 is abbreviated as not significant (n.s.).

Supplementary Figure 6 Rosetta-like locomotor activity patterns in 4 dpf *Dc*. Some 4 dpf *Dc* swim in what resembles concentric-like pathways (black) during the swimming **(A)**, first dark **(B)**, and first light **(C)** phase, resulting in Rosetta-like structures when depicted in a 300 s time interval; examples in **(A–C)** are from 3 different larvae. Such a peculiar locomotor activity was not observed in 4 dpf *AB* larvae [**D**; example pathway (black) during the swimming phase with a 300 s time interval as in **A–C**]. Each arena consist of a center (yellow) and outer (magenta) zone that are equal in area.

Supplementary Figure 7 Locomotor activity of 5 and 6 dpf *crystal* larvae in the light-dark test. **(A)** Locomotor activity of 5 dpf (green; *n* = 25) and 6 dpf (blue; *n* = 60) *crystal* larvae in the light-dark test; color-coded arrowheads highlight the increases in locomotor activity 1 s after the illumination switch. **(B,C)** Violin plots of the velocity during movement **(B)** and the time spent moving **(C)** for 5–6 dpf *crystal* larvae in the habituation (red) and swimming (blue) phase. **(D,E)** Violin plots of the velocity during movement **(D)** and the time spent moving **(E)** for 5–6 dpf *crystal* larvae in the light (yellow) and dark (gray) phases. Note the reduced time spent moving **(C,E)** of 5 dpf particularly during the habituation, swimming and light phases relative to 6 dpf *crystal* larvae. Two-way ANOVA followed by Šídák’s or Tukey’s multiple comparisons test was used to analyze differences in velocity or movement between phases of the light-dark test in and between 5 and 6 dpf *crystal*; *p* > 0.05 is abbreviated as not significant (n.s.).

Supplementary Figure 8 Startle responses evoked by illumination changes and thigmotaxis in 5 and 6 dpf *crystal* larvae. **(A)** Startle responses with standard error of the mean (SEM; shaded area) of 5 dpf (green, *n* = 25) and 6 dpf (blue, *n* = 60) *crystal* larvae depicted from 2 s before (2,398 s) to 18 s after (2,418 s) the first light to dark switch; a dotted black rectangle indicates the 1 s time interval that was used to compare the velocity of the larvae in **(B)**. **(B)** Violin plots depicting the velocity of 5 and 6 dpf *crystal* larvae during 1 s (2,400–2,401 s) following the first light (blue) to dark (gray) switch. **(C)** Startle responses of 5 and 6 dpf *crystal* larvae depicted 2 s before (2,998 s) and 18 s after (3,018 s) the first dark (gray) to light (yellow) switch; a dotted black rectangle indicates the 1 s time interval that was used to compare the velocity of the larvae in **(D)**. **(D)** Violin plots depicting the velocity of 5 and 6 dpf *crystal* larvae during 1 s (3,000–3,001 s) following the first dark to light switch. Student’s *t* or Mann–Whitney *U* test was used to analyze differences in velocity between 5 and 6 dpf *crystal*; *p* > 0.05 is abbreviated as not significant (n.s.). **(E,F)** Violin plots depicting the time spent in the outer zone of the wells show age-independent levels of thigmotaxis in 5 and 6 dpf *crystal* in all except the light phases of the test. Two-way ANOVA followed by Šídák’s or Tukey’s multiple comparisons test was used to analyze differences in thigmotaxis between phases of the light-dark test in and between 5 and 6 dpf *crystal*; *p* > 0.05 is abbreviated as not significant (n.s.).

Supplementary Figure 9 (Related to Supplementary Figures 8A–D). Startle responses evoked by illumination changes in 5 and 6 dpf *crystal* larvae. **(A)** Startle responses with standard error of the mean (SEM; shaded area) of 5 dpf (green, *n* = 25) and 6 dpf (blue, *n* = 60) *crystal* larvae depicted from 2 s before (3,598 s) to 18 s after (3,618 s) the second light to dark switch (compare with Supplementary Figure 8A); a dotted black rectangle indicates the 1 s time interval that was used to compare the velocity of the larvae in **(B)**. **(B)** Violin plots depicting the velocity of 5 and 6 dpf *crystal* larvae during 1 s (3,600–3,601 s) following the second light to dark switch (compare with Supplementary Figure 8B). **(C)** Startle responses of 5 and 6 dpf *crystal* larvae depicted 2 s before (4,198 s) and 18 s after (4,218 s) the second dark (gray) to light (yellow) switch; a dotted black rectangle indicates the 1 s time interval that was used to compare the velocity of the larvae in **(D)**. **(D)** Violin plots depicting the velocity of 5 and 6 dpf *crystal* larvae during 1 s (4,200–4,201 s) following the second dark to light switch. Mann–Whitney *U* test was used to analyze differences in velocity between 5 and 6 dpf *crystal*; *p* > 0.05 is abbreviated as not significant (n.s.).

Supplementary Figure 10 Body length of zebrafish and *Dc* larvae. **(A)** Violin plots depicting the body length of *AB* wildtype (green) and *crystal* (blue) zebrafish, and *Dc* (red) at 6 dpf show that both species are similar in size at this developmental age. **(B)** Violin plots depicting the body length of 4–6 dpf *Dc* show that 4 dpf (green) are significantly smaller than 5 dpf (blue) and 6 dpf (red) larvae. One-way ANOVA followed by Tukey’s multiple comparisons test was used to analyze differences in body length between *AB*, *crystal*, and *Dc*, and 4–6 dpf *Dc*; *p* > 0.05 is abbreviated as not significant (n.s.).
