# Supplementary figures and images for: A comparative analysis of Danionella cerebrum and zebrafish (Danio rerio) larval locomotor activity in a light-dark test
Source: Front Behav Neurosci. 2022 Aug 4;16:885775. doi: 10.3389/fnbeh.2022.885775 (PMC9385977; doi:10.3389/fnbeh.2022.885775)

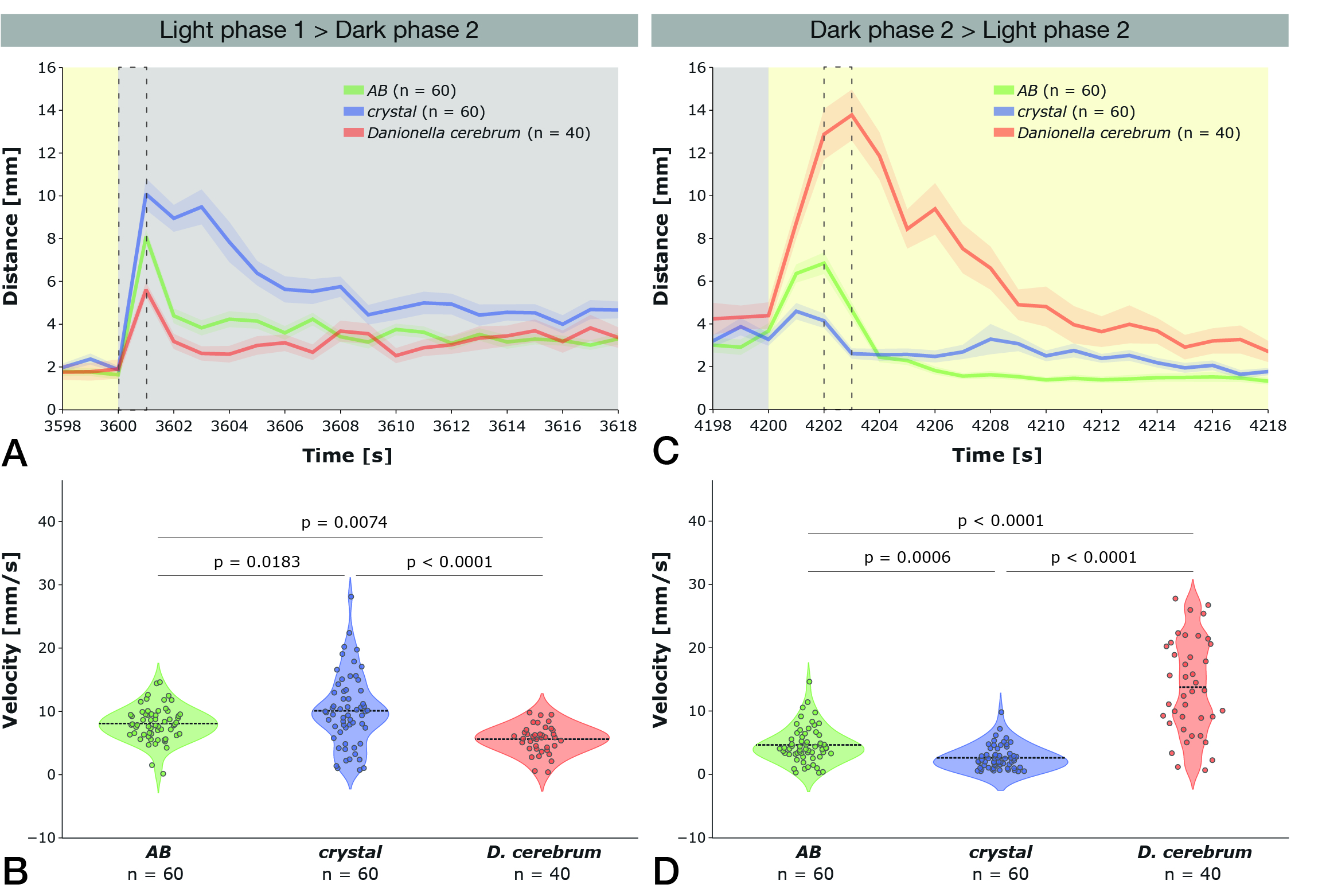

Supplement: Supplementary Figure 1 — (Related to Figures 2A–D). Different startle responses evoked by illumination changes in zebrafish and Dc larvae. (A) Startle responses ± SEM (shaded) of 6 dpf zebrafish AB wildtype (green; n = 60), crystal (blue; n = 60), and Dc (red; n = 40) larvae depicted from 2 s before (3,598 s) to 18 s after (3,618 s) the second light (yellow) to dark (gray) switch; a dotted black rectangle indicates the 1 s time interval that was used to compare the velocity of the larvae in (B). Note the similar responses of AB, crystal, and Dc larvae compared to the first light to dark switch depicted in Figure 2A. (B) Violin plots depicting the velocity of AB, crystal, and Dc larvae during 1 s (3,600–3,601 s) following the second light to dark switch (see also Figure 2B). (C) Startle responses of 6 dpf zebrafish AB wildtype (green), crystal (blue), and Dc (red) larvae depicted from 2 s before (4,198 s) to 18 s after (4,218 s) the second dark (gray) to light (yellow) switch; a dotted black rectangle indicates the 1 s time interval that was used to compare the velocity of the larvae in (D). Note the similar responses of AB, crystal, and Dc larvae compared to the first dark to light switch depicted in Figure 2C. (D) Violin plots depicting the velocity of AB, crystal, and Dc larvae during 1 s (4,202–4,203 s) following the second dark to light switch (see also Figure 2D). One-way ANOVA followed by Tukey’s multiple comparisons test or Kruskal–Wallis test followed by Dunn’s multiple comparisons test was used to analyze differences in velocity between AB, crystal, and Dc; p > 0.05 is abbreviated as not significant (n.s.). [file Image_1.JPEG]

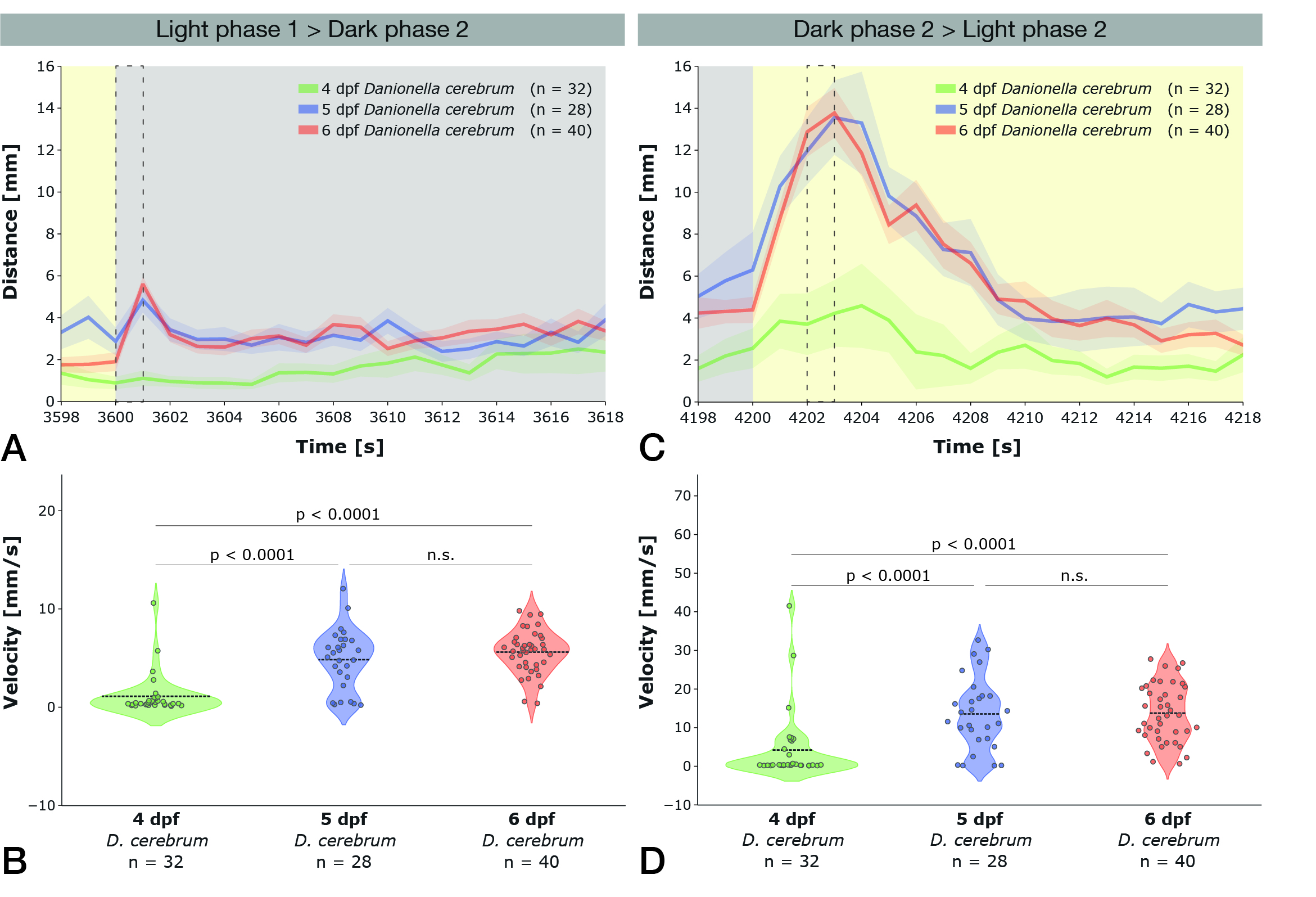

Supplement: Supplementary Figure 2 — (Related to Figures 4A–D). Age-dependent startle responses evoked by illumination changes in 4–6 dpf Dc larvae. (A) Startle responses with standard error of the mean (SEM; shaded area) of 4 dpf (green, n = 32), 5 dpf (blue, n = 28), and 6 dpf (red, n = 40) Dc larvae depicted from 2 s before (3,598 s) to 18 s after (3,618 s) the second light to dark switch (compare with Figure 4A); a dotted black rectangle indicates the 1 s time interval that was used to compare the velocity of the larvae in (B). (B) Violin plots depicting the velocity of 4–6 dpf Dc larvae during 1 s (3,600–3,601 s) following the second light to dark switch (compare with Figure 4B). (C) Startle responses of 4–6 dpf Dc larvae depicted 2 s before (4,198 s) and 18 s after (4,218 s) the second dark (gray) to light (yellow) switch; a dotted black rectangle indicates the 1 s time interval that was used to compare the velocity of the larvae in (D). (D) Violin plots depicting the velocity of 4–6 dpf Dc larvae during 1 s (4,202–4,203 s) following the second dark to light switch. Kruskal–Wallis test followed by Dunn’s multiple comparisons test was used to analyze differences in velocity between 4 and 6 dpf Dc; p > 0.05 is abbreviated as not significant (n.s.). [file Image_2.JPEG]

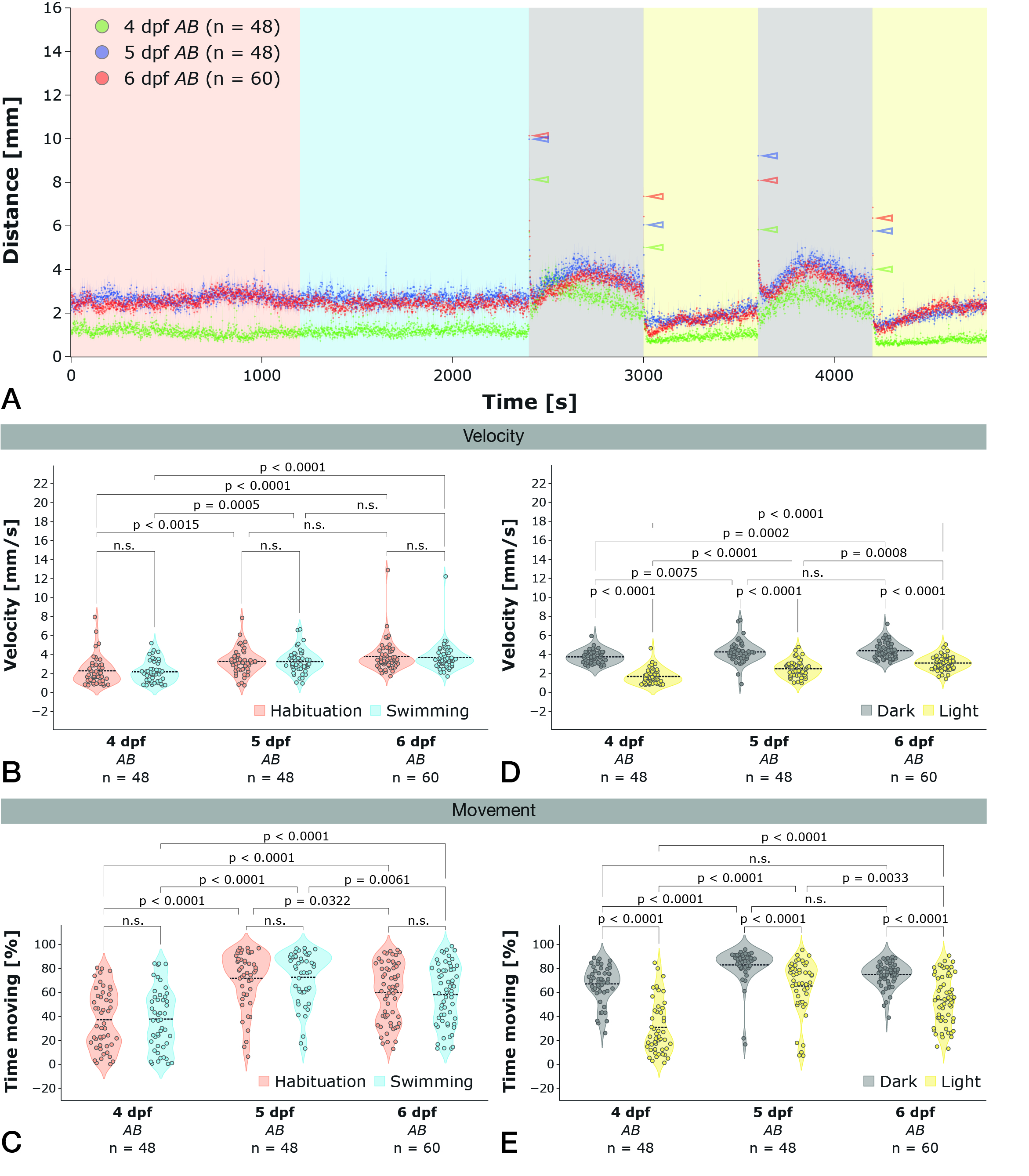

Supplement: Supplementary Figure 3 — Locomotor activity of 4–6 dpf AB larvae in the light-dark test. (A) Locomotor activity of 4 dpf (green; n = 48), 5 dpf (blue; n = 48), and 6 dpf (red; n = 60) AB larvae in the light-dark test; color-coded arrowheads highlight the increases in locomotor activity 1 s after the illumination switch. (B,C) Violin plots of the velocity during movement (B) and the time spent moving (C) for 4–6 dpf AB larvae in the habituation (red) and swimming (blue) phase. (D,E) Violin plots of the velocity during movement (D) and the time spent moving (E) for 4–6 dpf AB larvae in the light (yellow) and dark (gray) phases. Note the reduced velocity (B,D) and time spent moving (C,E) of 4 dpf particularly during the habituation, swimming and light phases relative to 5 and 6 dpf AB larvae. Two-way ANOVA followed by Šídák’s or Tukey’s multiple comparisons test was used to analyze differences in velocity or movement between phases of the light-dark test in and between 4 and 6 dpf AB; p > 0.05 is abbreviated as not significant (n.s.). [file Image_3.JPEG]

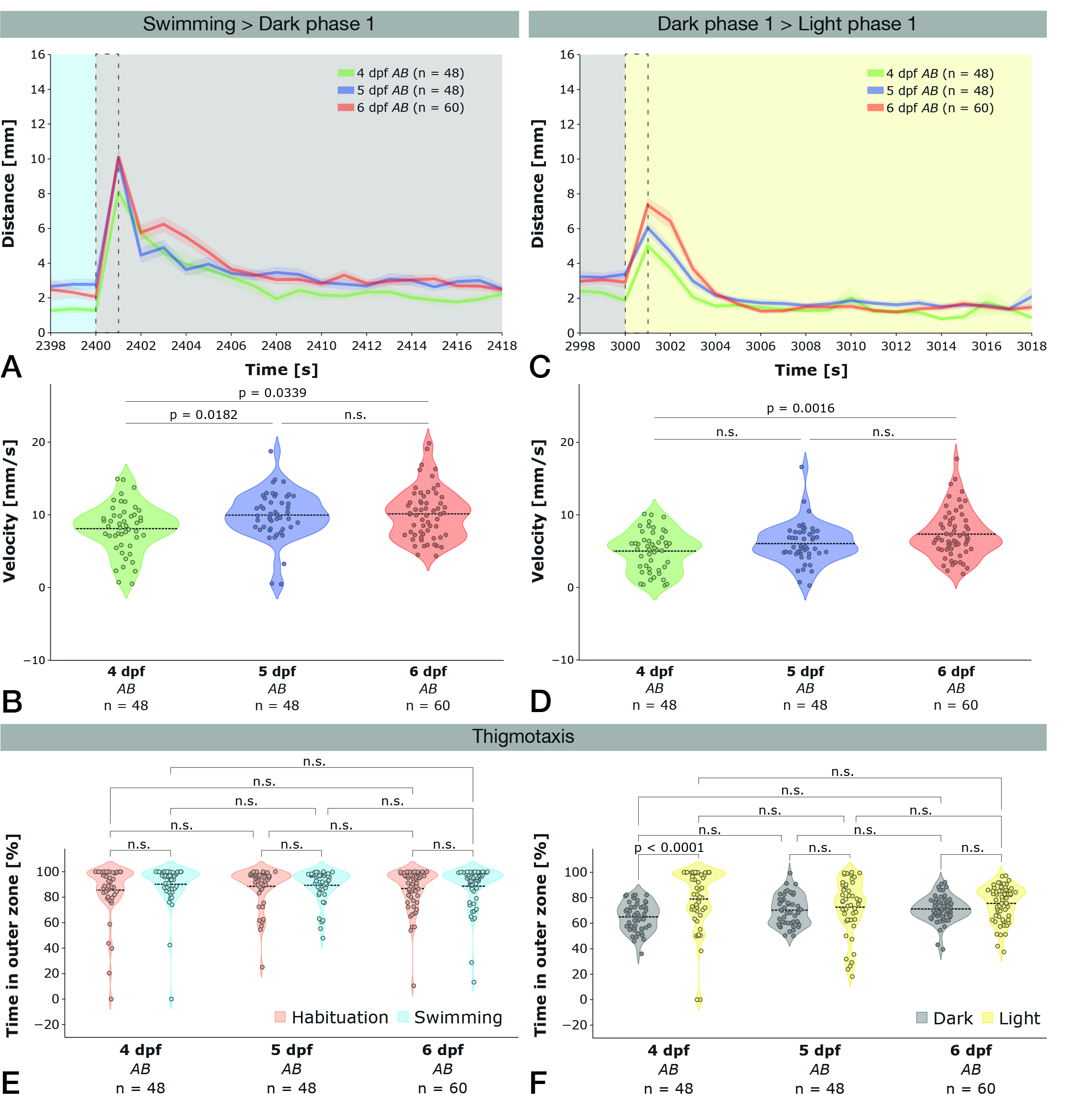

Supplement: Supplementary Figure 4 — Age-dependent startle responses evoked by illumination changes and age-independent thigmotaxis in 4–6 dpf AB larvae. (A) Startle responses with standard error of the mean (SEM; shaded area) of 4 dpf (green, n = 48), 5 dpf (blue, n = 48), and 6 dpf (red, n = 60) AB larvae depicted from 2 s before (2,398 s) to 18 s after (2,418 s) the first light to dark switch (see also Supplementary Figure 5A); a dotted black rectangle indicates the 1 s time interval that was used to compare the velocity of the larvae in (B). (B) Violin plots depicting the velocity of 4–6 dpf AB larvae during 1 s (2,400–2,401 s) following the first light to dark switch (see also Supplementary Figure 5B). (C) Startle responses of 4–6 dpf AB larvae depicted 2 s before (2,998 s) and 18 s after (3,018 s) the first dark (gray) to light (yellow) switch; (see also Supplementary Figure 5C); a dotted black rectangle indicates the 1 s time interval that was used to compare the velocity of the larvae in (D). (D) Violin plots depicting the velocity of 4–6 dpf AB larvae during 1 s (3,000–3,001 s) following the first dark to light switch (see also Supplementary Figure 5D). Note the relatively similar startle responses of 5 and 6 dpf AB larvae during the illumination changes. Kruskal–Wallis test followed by Dunn’s multiple comparisons test was used to analyze differences in velocity between 4 and 6 dpf AB; p > 0.05 is abbreviated as not significant (n.s.). (E,F) Violin plots depicting the time spent in the outer zone of the wells show age-independent levels of thigmotaxis in 4–6 dpf AB throughout all phases of the test and a significant decrease during the dark phases in 4 dpf (E). Two-way ANOVA followed by Šídák’s or Tukey’s multiple comparisons test was used to analyze differences in thigmotaxis between phases of the light-dark test in and between 4 and 6 dpf AB; p > 0.05 is abbreviated as not significant (n.s.). [file Image_4.JPEG]

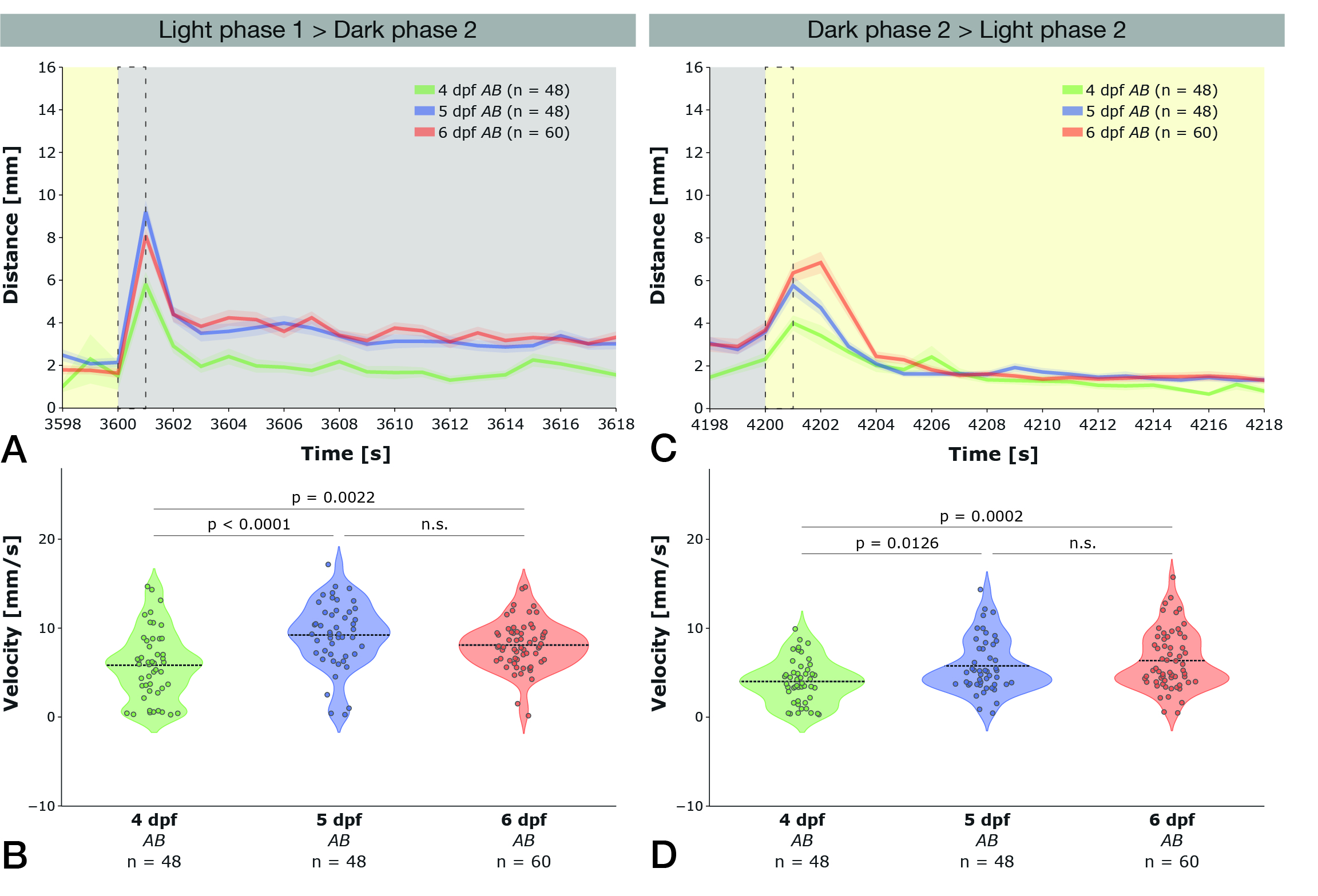

Supplement: Supplementary Figure 5 — (Related to Supplementary Figures 4A–D). Age-dependent startle responses evoked by illumination changes in 4–6 dpf AB larvae. (A) Startle responses with standard error of the mean (SEM; shaded area) of 4 dpf (green, n = 48), 5 dpf (blue, n = 48), and 6 dpf (red, n = 60) AB larvae depicted from 2 s before (3,598 s) to 18 s after (3,618 s) the second light to dark switch (compare with Supplementary Figure 4A); a dotted black rectangle indicates the 1 s time interval that was used to compare the velocity of the larvae in (B). (B) Violin plots depicting the velocity of 4–6 dpf AB larvae during 1 s (3,600–3,601 s) following the second light to dark switch (compare with Supplementary Figure 4B). (C) Startle responses of 4–6 dpf AB larvae depicted 2 s before (4,198 s) and 18 s after (4,218 s) the second dark (gray) to light (yellow) switch; a dotted black rectangle indicates the 1 s time interval that was used to compare the velocity of the larvae in (D). (D) Violin plots depicting the velocity of 4–6 dpf AB larvae during 1 s (4,200–4,201 s) following the second dark to light switch (see also Supplementary Figure 4D). Kruskal–Wallis test followed by Dunn’s multiple comparisons test was used to analyze differences in velocity between 4 and 6 dpf AB; p > 0.05 is abbreviated as not significant (n.s.). [file Image_5.JPEG]

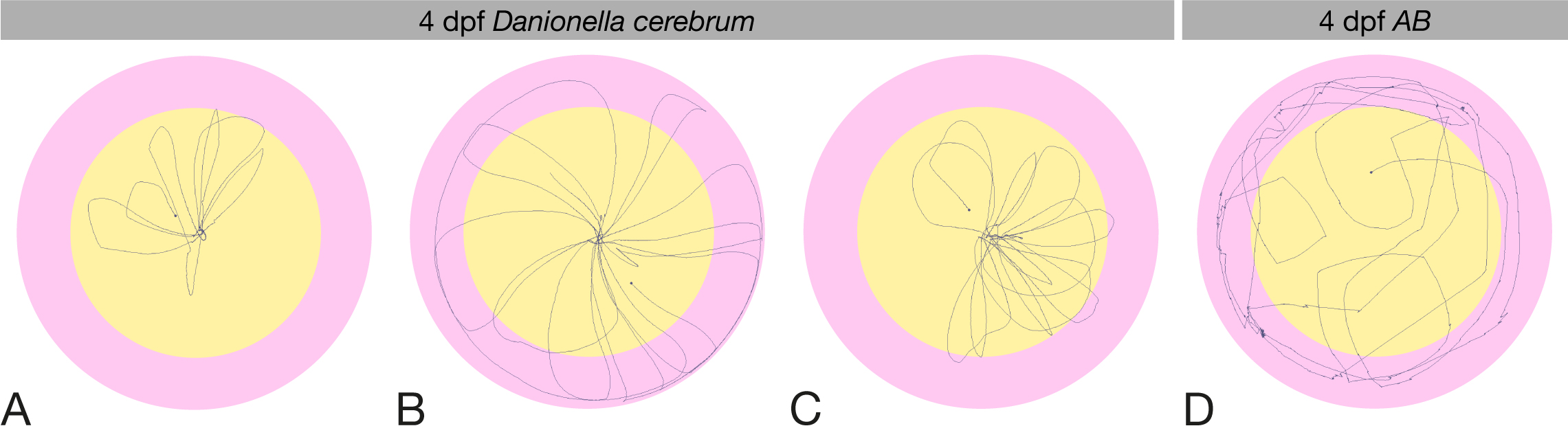

Supplement: Supplementary Figure 6 — Rosetta-like locomotor activity patterns in 4 dpf Dc. Some 4 dpf Dc swim in what resembles concentric-like pathways (black) during the swimming (A), first dark (B), and first light (C) phase, resulting in Rosetta-like structures when depicted in a 300 s time interval; examples in (A–C) are from 3 different larvae. Such a peculiar locomotor activity was not observed in 4 dpf AB larvae [D; example pathway (black) during the swimming phase with a 300 s time interval as in A–C]. Each arena consist of a center (yellow) and outer (magenta) zone that are equal in area. [file Image_6.JPEG]

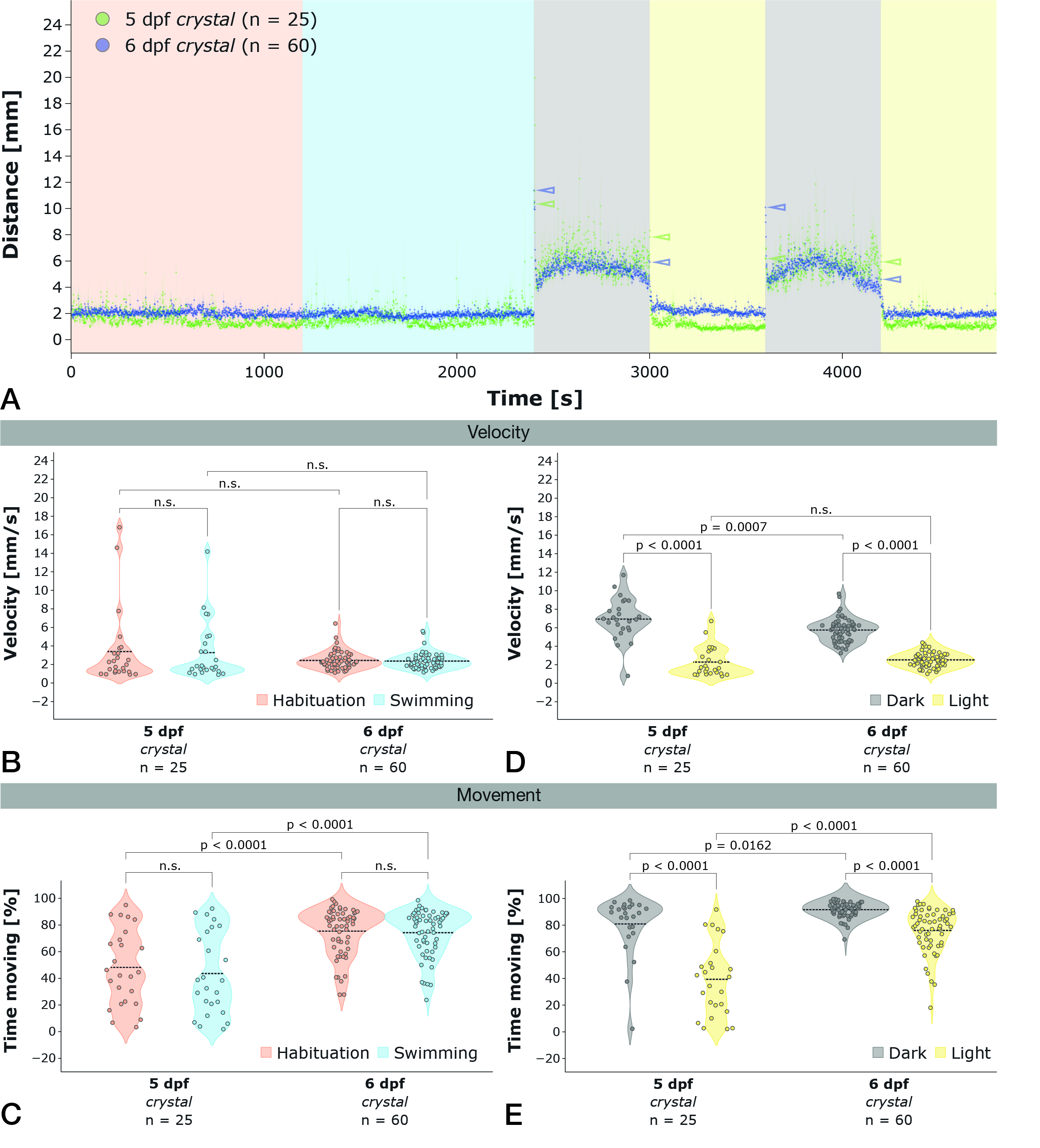

Supplement: Supplementary Figure 7 — Locomotor activity of 5 and 6 dpf crystal larvae in the light-dark test. (A) Locomotor activity of 5 dpf (green; n = 25) and 6 dpf (blue; n = 60) crystal larvae in the light-dark test; color-coded arrowheads highlight the increases in locomotor activity 1 s after the illumination switch. (B,C) Violin plots of the velocity during movement (B) and the time spent moving (C) for 5–6 dpf crystal larvae in the habituation (red) and swimming (blue) phase. (D,E) Violin plots of the velocity during movement (D) and the time spent moving (E) for 5–6 dpf crystal larvae in the light (yellow) and dark (gray) phases. Note the reduced time spent moving (C,E) of 5 dpf particularly during the habituation, swimming and light phases relative to 6 dpf crystal larvae. Two-way ANOVA followed by Šídák’s or Tukey’s multiple comparisons test was used to analyze differences in velocity or movement between phases of the light-dark test in and between 5 and 6 dpf crystal; p > 0.05 is abbreviated as not significant (n.s.). [file Image_7.JPEG]

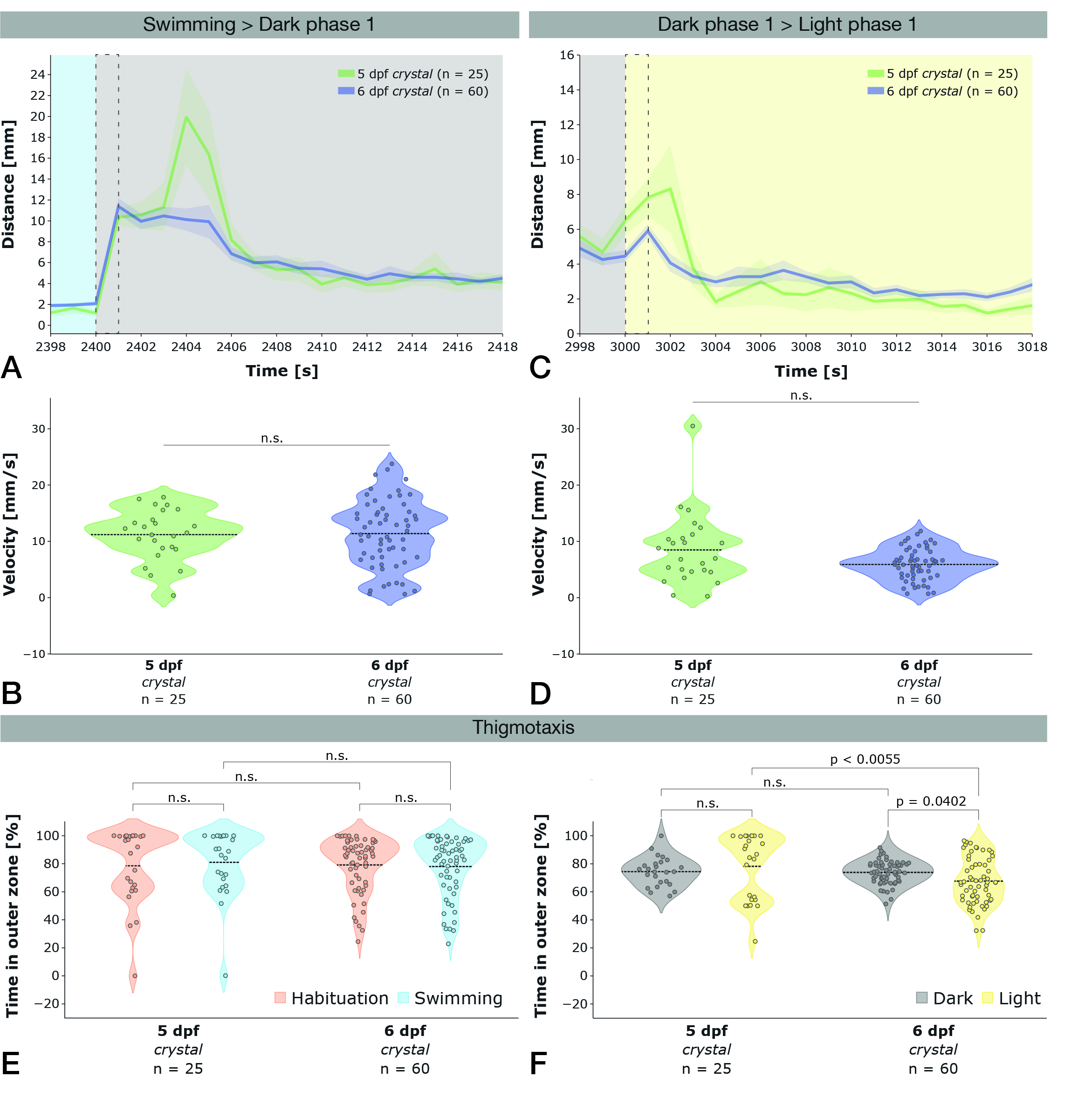

Supplement: Supplementary Figure 8 — Startle responses evoked by illumination changes and thigmotaxis in 5 and 6 dpf crystal larvae. (A) Startle responses with standard error of the mean (SEM; shaded area) of 5 dpf (green, n = 25) and 6 dpf (blue, n = 60) crystal larvae depicted from 2 s before (2,398 s) to 18 s after (2,418 s) the first light to dark switch; a dotted black rectangle indicates the 1 s time interval that was used to compare the velocity of the larvae in (B). (B) Violin plots depicting the velocity of 5 and 6 dpf crystal larvae during 1 s (2,400–2,401 s) following the first light (blue) to dark (gray) switch. (C) Startle responses of 5 and 6 dpf crystal larvae depicted 2 s before (2,998 s) and 18 s after (3,018 s) the first dark (gray) to light (yellow) switch; a dotted black rectangle indicates the 1 s time interval that was used to compare the velocity of the larvae in (D). (D) Violin plots depicting the velocity of 5 and 6 dpf crystal larvae during 1 s (3,000–3,001 s) following the first dark to light switch. Student’s t or Mann–Whitney U test was used to analyze differences in velocity between 5 and 6 dpf crystal; p > 0.05 is abbreviated as not significant (n.s.). (E,F) Violin plots depicting the time spent in the outer zone of the wells show age-independent levels of thigmotaxis in 5 and 6 dpf crystal in all except the light phases of the test. Two-way ANOVA followed by Šídák’s or Tukey’s multiple comparisons test was used to analyze differences in thigmotaxis between phases of the light-dark test in and between 5 and 6 dpf crystal; p > 0.05 is abbreviated as not significant (n.s.). [file Image_8.JPEG]

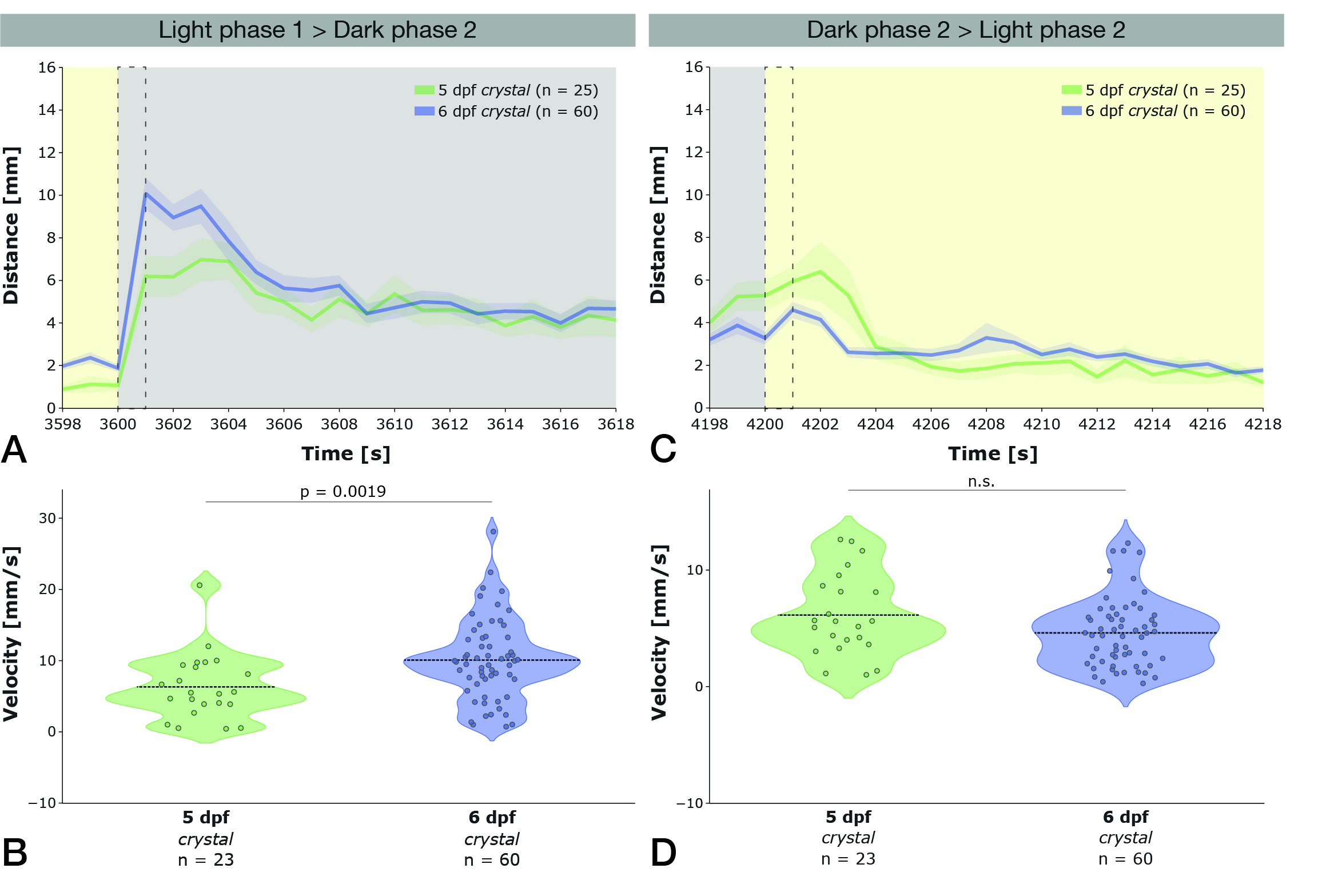

Supplement: Supplementary Figure 9 — (Related to Supplementary Figures 8A–D). Startle responses evoked by illumination changes in 5 and 6 dpf crystal larvae. (A) Startle responses with standard error of the mean (SEM; shaded area) of 5 dpf (green, n = 25) and 6 dpf (blue, n = 60) crystal larvae depicted from 2 s before (3,598 s) to 18 s after (3,618 s) the second light to dark switch (compare with Supplementary Figure 8A); a dotted black rectangle indicates the 1 s time interval that was used to compare the velocity of the larvae in (B). (B) Violin plots depicting the velocity of 5 and 6 dpf crystal larvae during 1 s (3,600–3,601 s) following the second light to dark switch (compare with Supplementary Figure 8B). (C) Startle responses of 5 and 6 dpf crystal larvae depicted 2 s before (4,198 s) and 18 s after (4,218 s) the second dark (gray) to light (yellow) switch; a dotted black rectangle indicates the 1 s time interval that was used to compare the velocity of the larvae in (D). (D) Violin plots depicting the velocity of 5 and 6 dpf crystal larvae during 1 s (4,200–4,201 s) following the second dark to light switch. Mann–Whitney U test was used to analyze differences in velocity between 5 and 6 dpf crystal; p > 0.05 is abbreviated as not significant (n.s.). [file Image_9.JPEG]

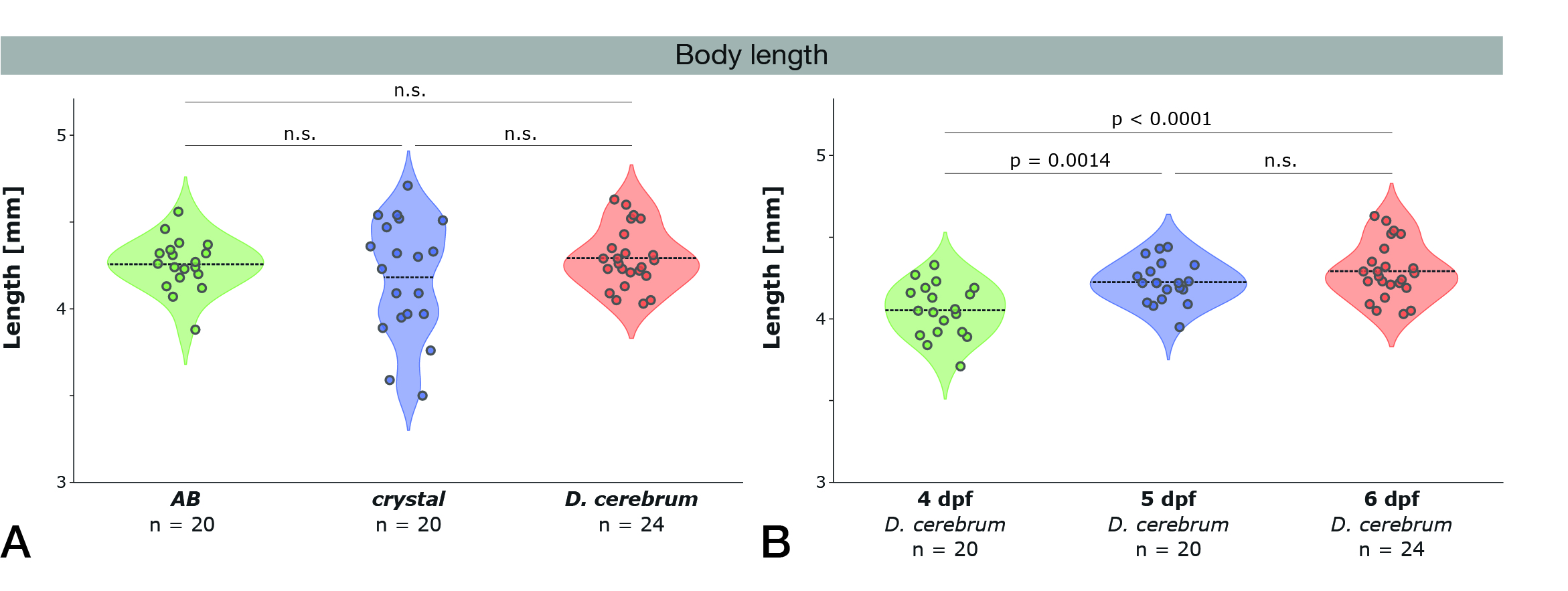

Supplement: Supplementary Figure 10 — Body length of zebrafish and Dc larvae. (A) Violin plots depicting the body length of AB wildtype (green) and crystal (blue) zebrafish, and Dc (red) at 6 dpf show that both species are similar in size at this developmental age. (B) Violin plots depicting the body length of 4–6 dpf Dc show that 4 dpf (green) are significantly smaller than 5 dpf (blue) and 6 dpf (red) larvae. One-way ANOVA followed by Tukey’s multiple comparisons test was used to analyze differences in body length between AB, crystal, and Dc, and 4–6 dpf Dc; p > 0.05 is abbreviated as not significant (n.s.). [file Image_10.JPEG]
